# Adipose tissue dysfunction as a central mechanism leading to dysmetabolic obesity triggered by chronic exposure to *p*,*p*’-DDE

**DOI:** 10.1038/s41598-017-02885-9

**Published:** 2017-06-01

**Authors:** Diogo Pestana, Diana Teixeira, Manuela Meireles, Cláudia Marques, Sónia Norberto, Carla Sá, Virgínia C. Fernandes, Luísa Correia-Sá, Ana Faria, Luísa Guardão, João T. Guimarães, Wendy N. Cooper, Ionel Sandovici, Valentina F. Domingues, Cristina Delerue-Matos, Rosário Monteiro, Miguel Constância, Conceição Calhau

**Affiliations:** 1 CINTESIS - Center for Health Technology and Services Research, Porto, Portugal; 2 Nutrition & Metabolism, NOVA Medical School|Faculdade de Ciências Médicas, Universidade NOVA de Lisboa, Lisboa, Portugal; 3 Department of Biochemistry, Faculty of Medicine, University of Porto, Porto, Portugal; 4 REQUIMTE/LAQV, Instituto Superior de Engenharia, Instituto Politécnico do Porto, Porto, Portugal; 5 REQUIMTE/LAQV, Department of Chemistry and Biochemistry, Faculty of Sciences, University of Porto, Porto, Portugal; 6 Animal House Department, Faculty of Medicine, University of Porto, Porto, Portugal; 7 Department of Clinical Pathology, Hospital S. João, Porto, Portugal; 8 University of Cambridge, Metabolic Research Laboratories, MRC Metabolic Diseases Unit, Department of Obstetrics & Gynaecology and National Institute for Health Research, Cambridge Biomedical Research Centre, Cambridge, UK; 9 Instituto de Investigação e Inovação em Saúde, Universidade do Porto, Porto, Portugal

## Abstract

Endocrine-disrupting chemicals such as *p*,*p*’-dichlorodiphenyldichloroethylene (*p*,*p*’-DDE), are bioaccumulated in the adipose tissue (AT) and have been implicated in the obesity and diabetes epidemic. Thus, it is hypothesized that *p*,*p*’-DDE exposure could aggravate the harm of an obesogenic context. We explored the effects of 12 weeks exposure in male Wistar rats’ metabolism and AT biology, assessing a range of metabolic, biochemical and histological parameters. *p*,*p*’-DDE -treatment exacerbated several of the metabolic syndrome-accompanying features induced by high-fat diet (HF), such as dyslipidaemia, glucose intolerance and hypertension. A transcriptome analysis comparing mesenteric visceral AT (vAT) of HF and HF/DDE groups revealed a decrease in expression of nervous system and tissue development-related genes, with special relevance for the neuropeptide *galanin* that also revealed DNA methylation changes at its promoter region. Additionally, we observed an increase in transcription of *dipeptidylpeptidase 4*, as well as a plasmatic increase of the pro-inflammatory cytokine IL-1β. Our results suggest that *p*,*p*’-DDE impairs vAT normal function and effectively decreases the dynamic response to energy surplus. We conclude that *p*,*p*’-DDE does not merely accumulate in fat, but may contribute significantly to the development of metabolic dysfunction and inflammation. Our findings reinforce their recognition as metabolism disrupting chemicals, even in non-obesogenic contexts.

## Introduction

A combination of complex events are contributing to the current obesity epidemic and ultimately to the metabolic syndrome (MetS), a serious worldwide health threat^1^. Metabolic disorders such as cardiovascular diseases (CVD) and type 2 diabetes mellitus (T2D) result from alterations of metabolic pathways in several organs. They are the result of the co-occurrence of obesity (particularly central obesity), dyslipidaemia, hyperglycaemia and hypertension^2^. Amongst obese individuals, variable prevalence of cardiometabolic complications is often observed, with metabolically healthy subjects accounting for 10–25%^3^. A central role in unhealthy obesity, also known as dysmetabolic obesity, is attributed to visceral adipose tissue (vAT) dysfunction and inflammation, affecting the dialog with other central (brain) and peripheral organs (liver, gut, muscle), but causal factors are still largely unknown^3, 4^.

The rate at which the prevalence of these conditions are increasing suggests that environmental and behavioural influences are “fuelling” the epidemic^5, 6^. Concerns have focused on the hypothesis that exposure to environmental endocrine-disrupting chemicals (EDCs) may be involved in the rise of obesity and MetS epidemic observed worldwide in the last 40 years. In particular, the man-made lipophilic persistent organic pollutants (POPs) with hormone-like activity (endocrine disruptors) can lead to an environmental disruption of metabolism and putative diabetogenic effect^5, 7–11^. POPs are bioaccumulated and biomagnified in the food chain and can be found in the adipose tissue (AT) of virtually all human populations^12^, functioning not only as a reservoir and a source of chronic internal exposure, but also as a possible tissue target of their disruptive effects^13, 14^. Due to their lipophilic nature, food consumption represents the main pathway for exposure to these contaminants, namely through ingestion of fat-containing food such as dairy products, meat and fish^8, 15–17^.

Recently, a series of studies concerning the burden of disease and cost analysis of exposure to EDCs revealed a likely substantial contribution to disease and dysfunction across the life course with elevated annual costs, both in the European Union and USA, emphasizing the need for evaluation of effects, better monitoring and active prevention^18–20^. Supporting these warnings are, among others, several epidemiological and *in vivo* studies that reported the positive correlation of EDCs concentrations, both in AT and plasma, with the prevalence of obesity, MetS, insulin resistance, hypertension and CVD^21–23^, as well as adipocyte dysfunction^24^.

Studies such as those developed by Ruzzin *et al*. where dietary supplementation with salmon oil contaminated with a mixture of POPs in conjunction with high-fat (HF) diet feeding promoted the development of obesity and T2D in a rodent model^25, 26^, led to the proposition of a broader metabolism disrupting chemical hypothesis where ECDs act as metabolic disruptors that increase the susceptibility to metabolic diseases^7, 10, 27, 28^. A recent publication reviewed the putative mechanisms of metabolic disruption^28^, highlighting the effects on adipogenesis and adipokine production^29, 30^, neuroendocrine control of feeding and metabolism^31, 32^, energy homeostasis and T2D trough alteration of insulin action, glucose disposal and beta cell survival and function^33^, as well as hepatic steatosis^34^, and hyperlipidemia^35^. Involved molecular mechanism encompass the alteration of hormonal and homeostatic systems related to sex hormones (e.g. estrogens and androgens, thyroid function and corticosteroids) acting through nuclear receptors, nonnuclear steroid hormone receptors, nonsteroid receptors (for example, neurotransmitter receptors), orphan receptors (such as aryl hydrocarbon receptor, AhR), endoplasmic reticulum and oxidative stress, inflammatory disruption and epigenetic changes^7, 28^.

In a recent paper, Gauthier *et al*.^36^ showed that the metabolically dysfunctional obese phenotype was associated with higher plasma POP levels. Results from our group in obese individuals revealed that POP levels especially in vAT, the main reservoir, were higher in subjects with evidence of metabolic abnormalities^27, 37^, thus complementing and strengthening the Gauthier study. In this context, the mechanisms by which POPs exert their effects could involve various pathways including dysregulation of hormonal systems, nuclear receptors and epigenetic processes, in addition to possible action on neurotransmitter receptors and systems^38^. Altogether, these mechanisms could ultimately interfere with AT remodeling and function with local and systemic consequences^21, 24, 39^. Hence, POPs stored in AT may be a key component in the pathogenesis of MetS, in synergy with lifestyle factors, to promote obesity and its associated complications, namely T2D^21, 24, 40^.

One of these synthetic organic chemicals is the pesticide *p*,*p*’-dichlorodiphenyldichloroethylene (*p*,*p*’-DDE), which has been associated with an increased prevalence of obesity and T2D in several epidemiological studies^41, 42^. However the causal relationship and specific mechanisms of action are still under discussion. This study was designed to evaluate if exposure of rats to sustained low levels of *p*,*p*’-DDE (2.5 times less than lowest-observed-adverse-effect level), either in presence or absence of a HF diet, could contribute to the appearance or aggravation of metabolic disorders and possible functioning as an environmental modifier between obesity subphenotypes^3^. For this purpose, the metabolic characterization of these rats was accompanied by a comprehensive and integrative evaluation of vAT histology, transcriptome and DNA methylation changes in key differentially expressed genes.

## Results

### Weight measurements and body composition

**Figure 1:**
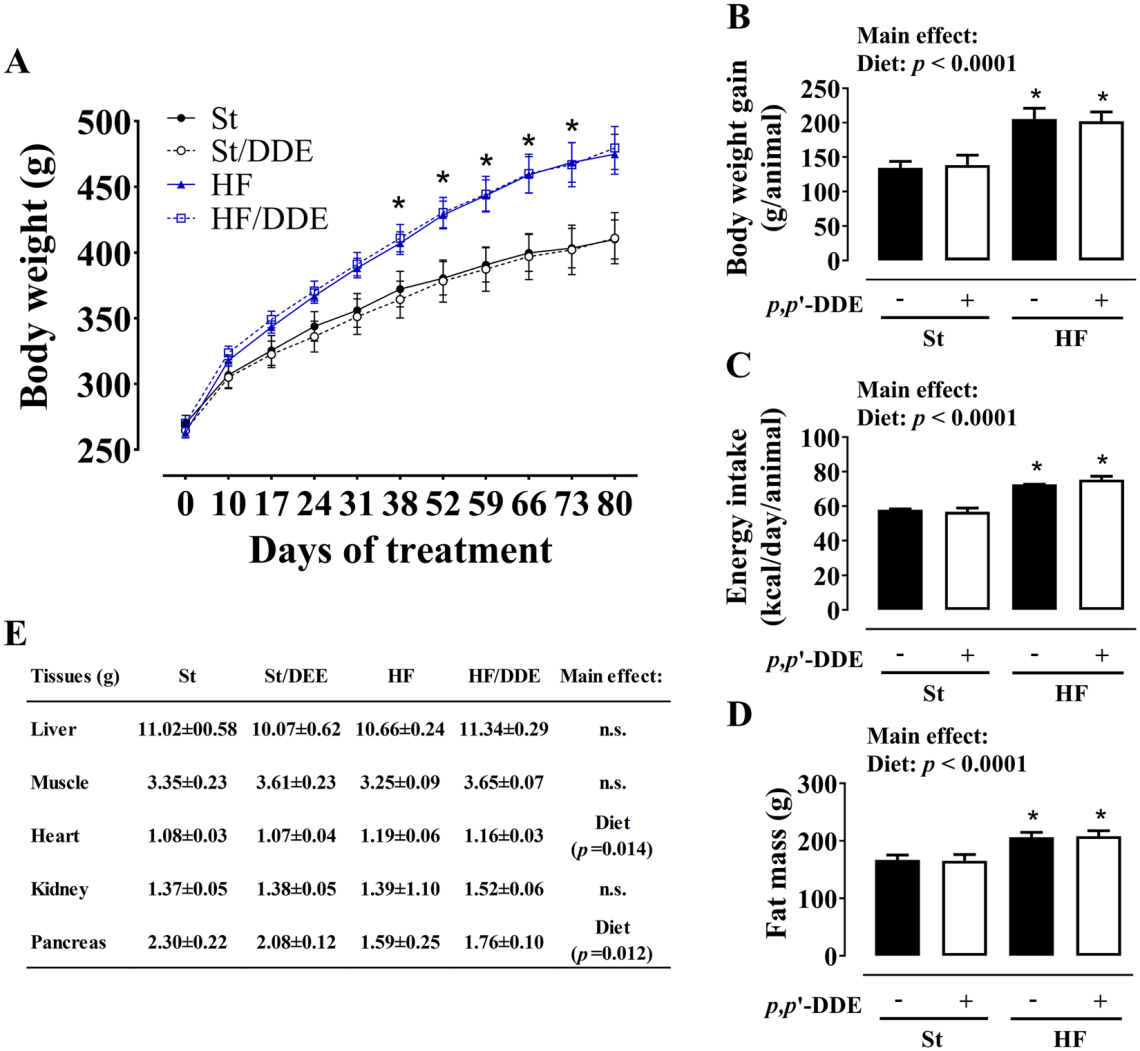
Weight measurements and body composition: (**A**) body weight evolution throughout the 12 weeks of treatment; (**B**) body weight gain per animal; (**C**) energy intake (kcal) per day/animal calculated trough food intake assessment; (**D**) fat mass evaluated by bioelectrical impedance at 12 weeks; (**E**) organ weights at 12 weeks. Values are represented as mean ± SEM. Statistical analysis with (**A**) two-way repeated-measures ANOVA; (**B**,**C**,**D** and **F**) two-way ANOVA (main effects: diet, *p*,*p*’-DDE exposure and their interaction; *p* < 0.05), followed by Tukey’s multiple comparison post-hoc test: *p < 0.05 vs St and St/DDE. St, standard diet; HF, high fat diet; *p*,*p*’-DDE, *p*,*p*’-dichlorodiphenyldichloroethylene.

HF fed rats had a significantly accelerated growth during the treatment (Fig. 1), evident in body weight evolution, weight gain per animal and energy intake (kcal/day/animal). Exposure to *p*,*p*’-DDE did not alter body weight profile in any of the diets. At the end of the treatment, both groups that ingested HF had significantly higher fat mass (bioelectrical impedance evaluation, *p* < 0.0001) and an effect in heart and pancreas (*p* = 0.014 and *p* = 0.012), respectively increasing and decreasing their weights.

### AT - distribution, lipolysis and *p*,*p*’-DDE accumulation

**Figure 2:**
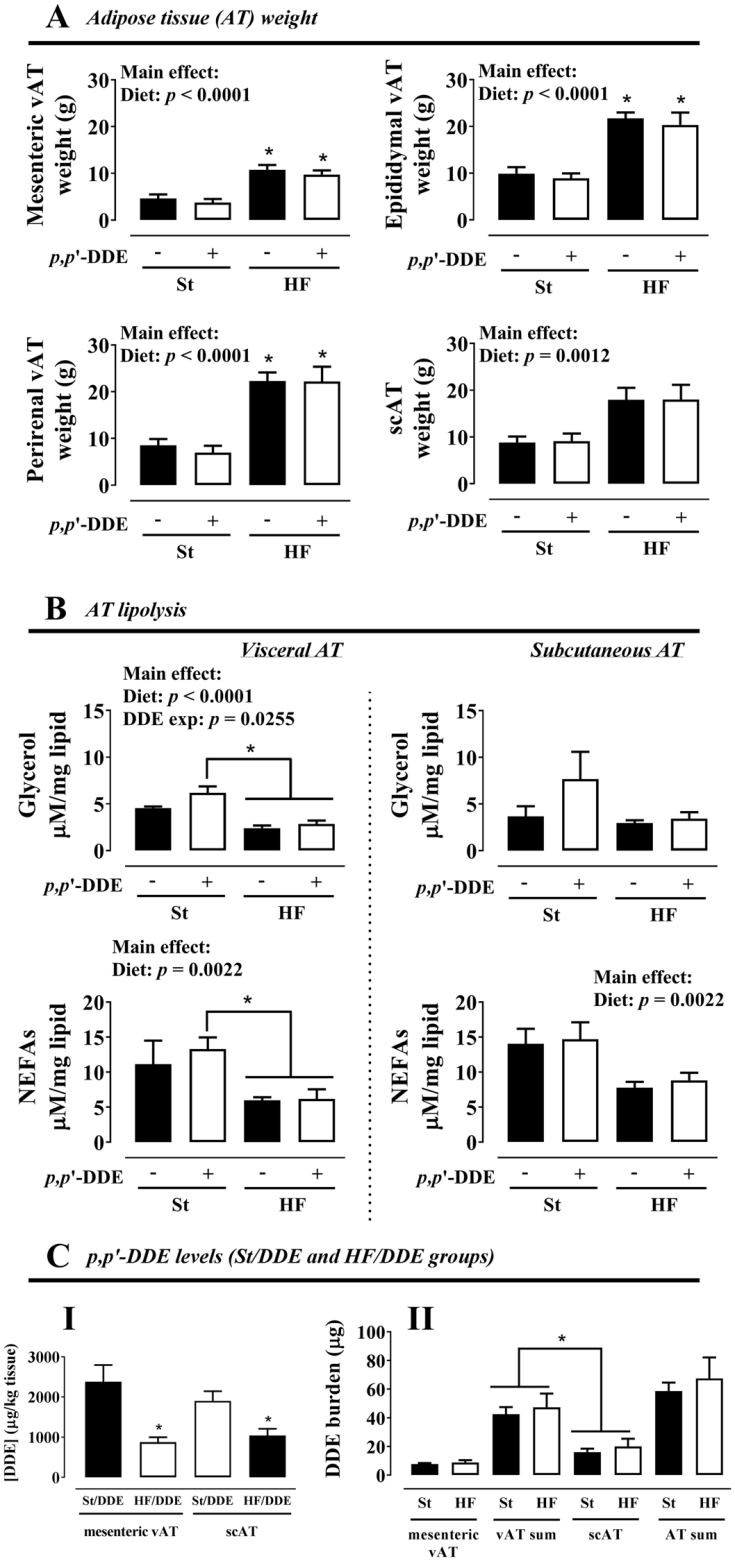
Adipose tissue (AT) depot weight (**A**), lipolysis in isolated mature adipocytes (**B**) and *p*,*p*’-DDE levels in AT (concentration, **CI**; burden, **CII**), after 12 weeks. Values are represented as mean ± SEM. Statistical analysis with (**A**,**B**) two-way ANOVA (main effects: diet, *p*,*p*’-DDE exposure and their interaction; *p* < 0.05), followed by Tukey’s multiple comparison post-hoc test: *p < 0.05 *vs* St and/or St/DDE; (**C**) unpaired *t* test when comparing between treatment groups (St/DDE and HF/DDE) and paired *t* test when comparing between different ATs from the same treatment group: *p < 0.05. St, standard diet; HF, high fat diet; *p*,*p*’-DDE, *p*,*p*’-dichlorodiphenyldichloroethylene; vAT, visceral adipose tissue; scAT, subcutaneous adipose tissue; NEFAs, non-esterified fatty acids.

HF diet, independently of exposure to *p*,*p*’-DDE, had a significant effect on AT weight increase (Fig. 2A). In contrast, vAT mature adipocytes basal lipolysis (Fig. 2B) was decreased when *p*,*p*’-DDE exposed and non-exposed animals were fed with HF diet, translated by a smaller glycerol and non-esterified fatty acids (NEFAs) release (*p* < 0.0001 and *p* = 0.0022). Nevertheless, *p*,*p*’-DDE exposure increased vAT lipolysis (glycerol, *p* = 0.0255), an effect more pronounced in St/DDE rats without the overwhelming effect of HF diet. *p*,*p*’-DDE was only present in AT of exposed rats (data not shown) and its concentration was higher in St/DDE compared to HF/DDE rats in both mesenteric and subcutaneous AT depots (Fig. 2CI). However, no difference between exposed groups was observed in *p*,*p*’-DDE burden, calculated after normalization for AT weight (Fig. 2CII).

### Evaluation of MetS features

**Figure 3:**
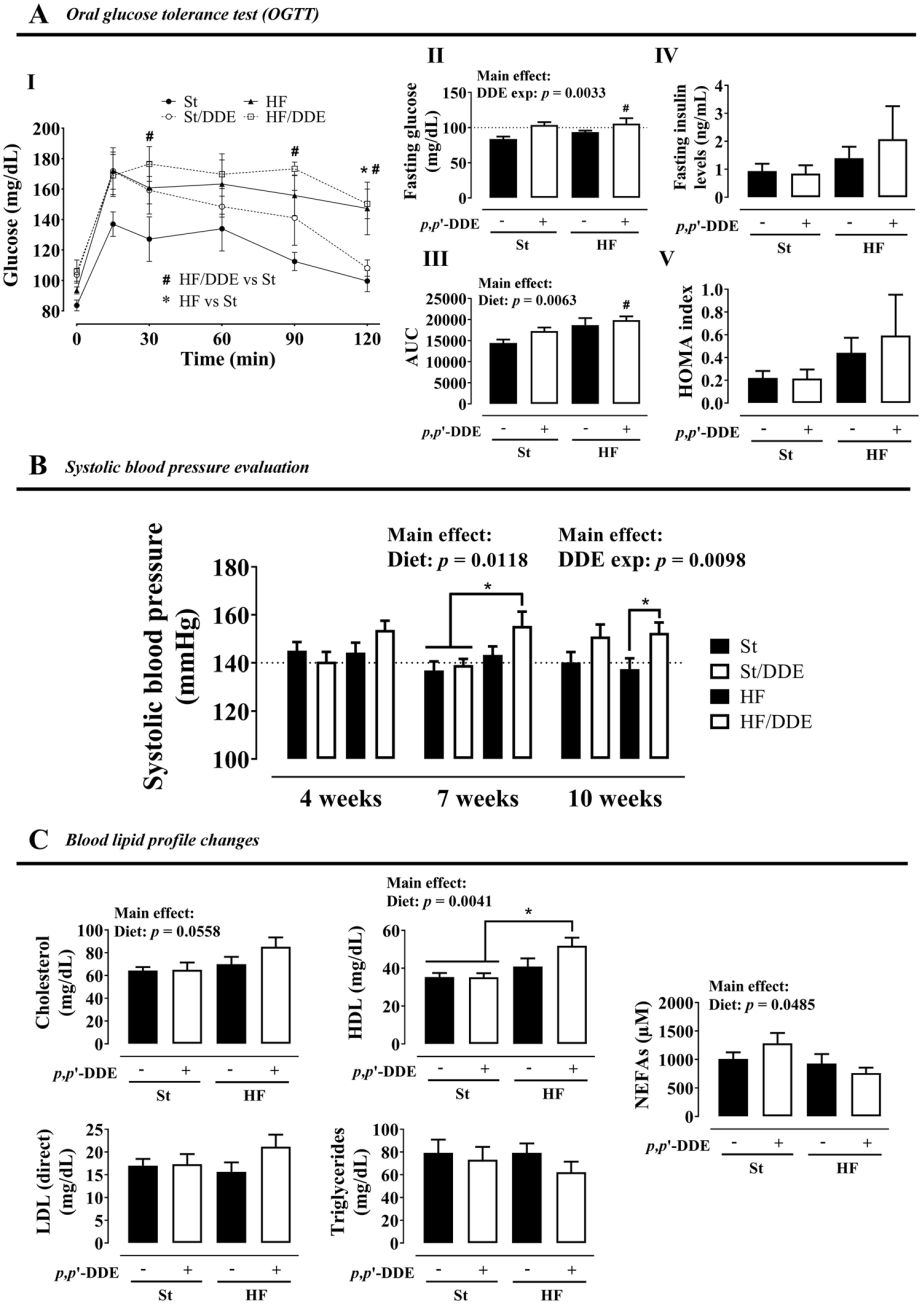
Metabolic syndrome features. (**A**) oral glucose tolerance test (OGTT) at the 7th week: (**I**) blood glucose variation, (**II**) fasting glucose, (**III**) blood glucose area under the curve (AUC) and (**IV**) fasting insulin levels and (**V**) homeostasis model assessment (HOMA) of insulin resistance after 12 weeks; (**B**) systolic blood pressure at 4, 7 and 10 weeks; and (**C**) plasma lipid profile at the end of treatment: total cholesterol, triglycerides, high-density lipoprotein (HDL) cholesterol, cholesterol low-density lipoprotein (LDL) and non-esterified fatty acids (NEFAs). Statistical analysis with two-way ANOVA (main effects: diet, *p*,*p*’-DDE exposure and their interaction; *p* < 0.05), followed by Tukeys’s multiple comparison post-hoc test. (**A**) ^#^ *p* < 0.05 HF/DDE *vs* St and *p < 0.05 HF *vs* St; (**B**,**C**) *p < 0.05 between selected groups. St, standard diet; HF, high fat diet; *p*,*p*’-DDE, *p*,*p*’-dichlorodiphenyldichloroethylene.

An oral glucose tolerance test (OGTT) was performed after 7 weeks of treatment (Fig. 3A) to evaluate the effects of *p*,*p*’-DDE exposure in glycaemic response in both diets. The animals from both groups fed with HF diet exhibited impaired glucose tolerance, being more pronounced in HF/DDE group (Fig. 3AI). Unlike standard diet-fed groups, glycaemia did not return to baseline in HF/DDE and HF after 120 min of glucose administration. Total area under the curve (AUC) of the glycaemic response was calculated for each experimental group (Fig. 3AIII). HF diet increased the AUC independently of *p*,*p*’-DDE exposure (*p* = 0.0063), but in addition to a later and higher glucose peak in the OGTT, the effect of HF/DDE treatment was also reflected by a higher AUC comparing to St (*p* < 0.05, Tukey’s post-hoc test). In turn, *p*,*p*’-DDE exposure had a significant increasing effect on fasting glucose independently of the diet (*p* = 0.0033, Fig. 3AII). Fasting insulin levels (Fig. 3AIV) at the end of the treatment were tendentiously higher in HF diet groups, a tendency more pronounced in HF/DDE animals, which is in accordance to the results obtained for this group in OGTT. Correspondingly, homeostasis model assessment (HOMA) of insulin resistance indicated the same pattern (Fig. 3AV).

HF/DDE rats were hypertensive since week 4, with systolic blood pressure (SBP) >140 mmHg (Fig. 3B). Overall, *p*,*p*’-DDE exposure was responsible for SBP variation, demonstrated by higher SBP in St/DDE and HF/DDE at 10 weeks (*p* = 0.0098). At the end of treatment (Fig. 3C), we observed that high-density lipoprotein (HDL) cholesterol levels were higher in animals fed with HF diet (p = 0.0041), however the increase in HDL levels caused by HF/DDE treatment was more pronounced comparing to St groups (*p* < 0.05, Tukey’s post-hoc test). The same pattern was observed for cholesterol. Regarding NEFAs, the HF diet effect was opposite to HDL, with lower levels in HF-treated animals (*p* = 0.0485).

### Biochemical parameters and circulating cytokine profiles

Plasma and urine biochemical parameters can be found as Supplementary Table S1. A significant interaction between HF diet and *p*,*p*’-DDE exposure was observed, resulting in the increase of plasma uric acid and lactate levels only in HF/DDE animals (interaction; *p* = 0.0015 and *p* = 0.0114). The same pattern was observed in some of the cellular damage plasma marker levels, with a significant increase of amylase, aspartate aminotransferase/alanine aminotransferase (AST/ALT) ratio, AST and creatine kinase (CK) only in HF/DDE animals (interaction; *p* = 0.0156, *p* = 0.0376, *p* = 0.0484 and *p* = 0.0241, respectively).

Urinary glucose levels were higher in HF diet-fed groups (*p* = 0.0301), but more prominent in HF/DDE animals. In the same way, microalbuminuria, which presence in urine is in itself alarming, was affected both by diet and *p*,*p*’-DDE exposure (*p* = 0.0473 and *p* = 0.0332, respectively), with a significant increase in HF/DDE animals compared to their St and HF counterparts (*p* < 0.05, Tukey’s post-hoc test).

**Figure 4:**
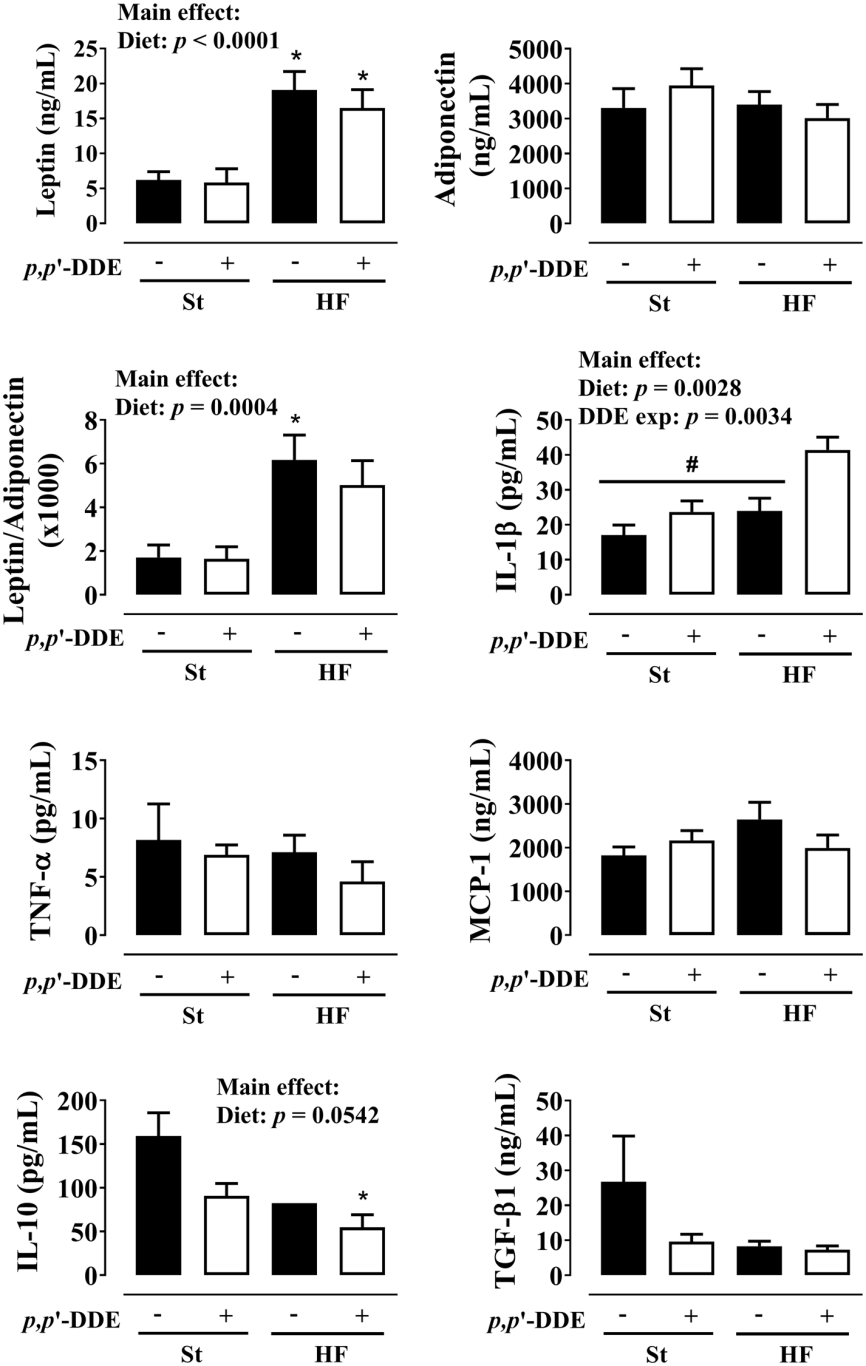
Effect of 12 weeks of treatment on circulating cytokine profile: leptin, adiponectin and their ratio; pro-inflammatory cytokines interleukin-1 *beta* (IL-1β), tumor necrosis factor-*alpha* (TNF-α) and monocyte chemotactic protein 1 (MCP-1); anti-inflammatory cytokines interleukin-10 (IL-10) and transforming growth factor-*beta* 1 (TGF-β1). Statistical analysis with two-way ANOVA (main effects: diet, *p*,*p*’-DDE exposure and their interaction; *p* < 0.05), followed by Tukey’s multiple comparison post-hoc test: *p < 0.05 *vs* St; ^#^ *p* < 0.05 *vs* HF/DDE. St, standard diet; HF, high fat diet; *p*,*p*’-DDE, *p*,*p*’-dichlorodiphenyldichloroethylene.

Circulating cytokines levels at 12 weeks (Fig. 4), showed a significant increase in leptin and leptin/adiponectin ratio by HF diet (*p* < 0.0001 and *p* = 0.0004). No significant differences were observed in TNF-α and MCP-1 levels. In contrast, IL-1β levels increased both by HF (*p* = 0.0028) and *p*,*p*’-DDE exposure (*p* = 0.0034), but with a significant increase in HF/DDE-treated rats when compared with all other groups (*p* < 0.05, Tukey’s). We observed an opposite effect for anti-inflammatory cytokines IL-10 and TGF-β1.

### Mesenteric vAT morphology

**Figure 5:**
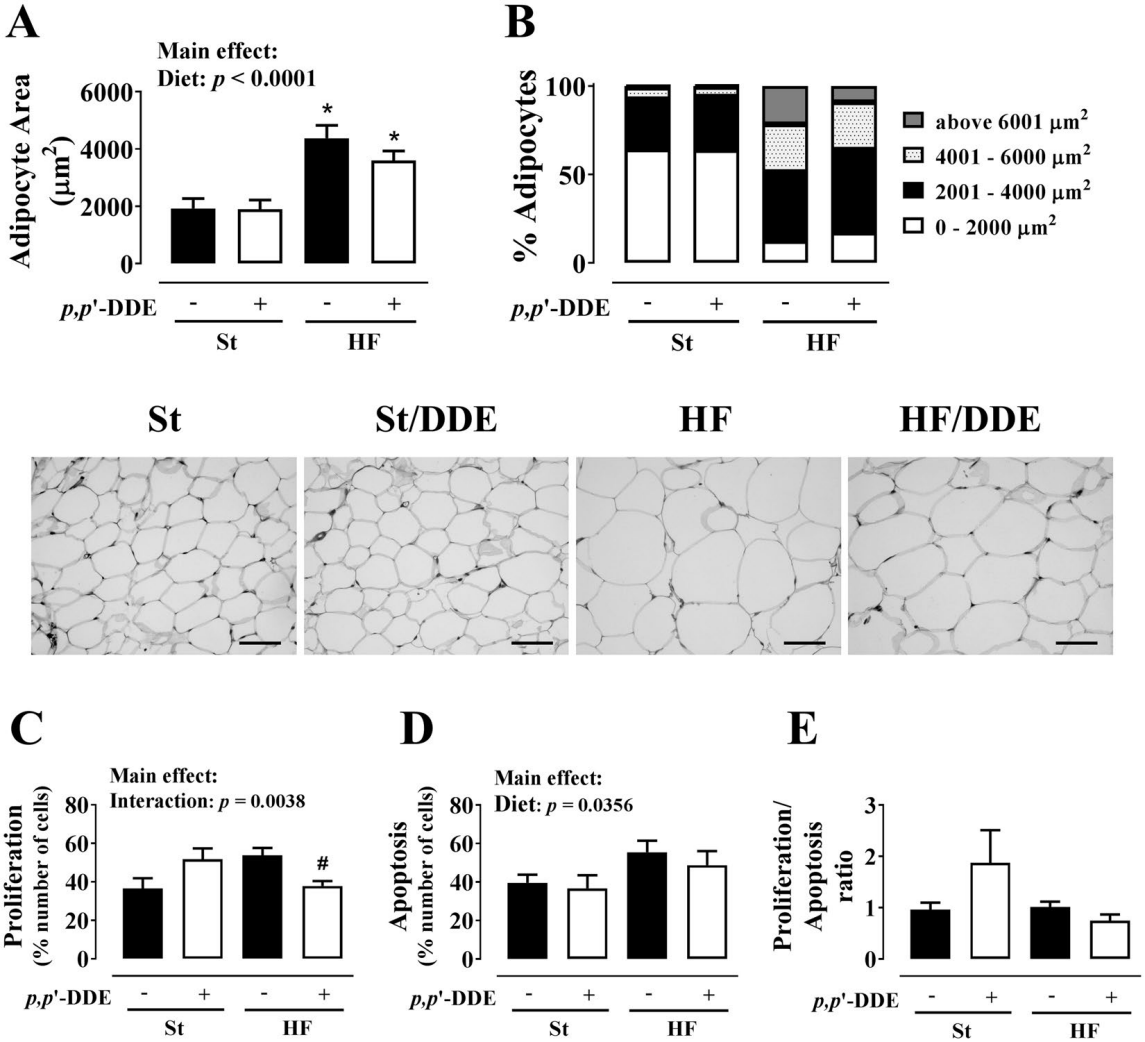
Mesenteric visceral adipose tissue morphology and proliferative and apoptotic status. (**A**) adipocyte area; (**B**) adipocyte distribution regarding their size; (**C**) proliferation; (**D**) apoptosis; (**E**) proliferation/apoptosis ratio. Statistical analysis with two-way ANOVA (main effects: diet, *p*,*p*’-DDE exposure and their interaction; *p* < 0.05), followed by Tukey’s multiple comparison post-hoc test: *p < 0.05 *vs* St; ^#^ *p* < 0.05 *vs* HF. Scale bar represents 200 μm. St, standard diet; HF, high fat diet; *p*,*p*’-DDE, *p*,*p*’-dichlorodiphenyldichloroethylene.

Adipocyte area (Fig. 5) was higher in HF diet groups (*p* < 0.0001). HF/DDE rats (*vs* HF) had an increase in % smaller/medium (<4000 µm^2^) and a decrease of bigger (>6000 µm^2^) adipocytes. We observed increased proliferation in St/DDE and HF rats, but the interaction between HF diet and *p*,*p*’-DDE exposure led to a significant decrease (*p* = 0.0038). Moreover, HF diet contributed for increased percentage of apoptotic cells, independently of exposure to *p*,*p*’-DDE (*p* = 0.0356).

### Comparison of global gene expression between HF and HF/DDE rats

Gene expression microarrays in mesenteric vAT from HF and HF/DDE rats showed that 320 genes were up-regulated and 311 were down-regulated (at least 1.25-fold change). Detailed data concerning up- and down-regulated genes are shown in Supplementary Table S2. The largest changes induced by HF/DDE were seen in down-regulated genes, such as galanin (*Gal*; −4.92-fold), cholinergic receptor nicotinic *alpha* 3 (*Chrna3*; −3.20-fold), somatostatin (*Sst*; −2.88-fold), neuromedin U (*Nmu*; −2.75-fold) or neuropeptide Y (*Npy*; −1.78-fold). Expression of genes such as dipeptidylpeptidase 4 (*Dpp4*; 2.36-fold) and ISL LIM homeobox 1 (*Isl1*; 1.77-fold) were up-regulated.

The differentially transcribed genes, mainly the down-regulated genes, were associated with several biological functions and pathways (Table 1). Cell-to-cell signalling and interaction, nervous system development and function, molecular transport, cellular assembly, organization and function, as well as cell and tissue morphology were on the top down-regulated functions by HF/DDE. Top transcription factors associated with HF/DDE (see Supplementary Fig. S1) disclosed an activation of hepatocyte nuclear factor 4 *alpha* (*HNF4A*; z-score 2.427) and a contrary trend for estrogen receptor *alpha* (*Esr1*). Simulating a data network between transcription factors and modulated genes using IPA, we observed a link between the transcription factors *Esr1* and *Isl1*, and *Gal*.

**Table 1:** Ingenuity pathway analysis of HF/DDE regulated genes, either evaluating the overall differentially transcribed genes or with down and up-regulated genes analysed in separate. The ratio refers to the proportion of molecules within the pathway.

Top overall Bio functions and pathways			Ranking of functions (down and up-regulated genes analysed in separate)			
Molecular and Cellular Functions	*p* value	# genes	Down-regulated genes	*p* value	Up-regulated genes	*p* value
Cell-to-cell Signalling and interaction	1.93 × 10^−12^	75	Cell-to-cell Signalling and interaction	1.96 × 10^−25^	Tissue Morphology	2.04 × 10^−4^
Molecular transport	5.67 × 10^−10^	68	Nervous System Development and Function	1.21 × 10^−18^	DNA Replication, Recombination, and Repair	2.68 × 10^−4^
Small molecule biochemistry	5.67 × 10^−10^	75	Genetic Disorder	1.62 × 10^−17^	Cellular Assembly and Organization	4.71 × 10^−4^
Cell morphology	5.40 × 10^−07^	56	Neurological Disease	1.62 × 10^−17^	Cellular Function and Maintenance	4.71 × 10^−4^
Cellular assembly and organization	5.40 × 10^−07^	95	Psychological Disorders	1.62 × 10^−17^	Cellular Development	6.62 × 10^−4^
**Physiological system development and function**	***p*** **value**	**# genes**	Behaviour	1.65 × 10^−12^	Embryonic Development	6.62 × 10^−4^
Nervous system development and function	1.93 × 10^−12^	114	Skeletal and Muscular Disorders	7.28 × 10^−12^	Gene Expression	7.71 × 10^−4^
Behaviour	2.26 × 10^−07^	59	Molecular Transport	9.57 × 10^−10^	Cancer	1.31 × 10^−3^
Tissue development	4.06 × 10^−06^	56	Small Molecule Biochemistry	9.57 × 10^−10^	Cell Death	1.31 × 10^−3^
Skeletal and muscular system development and function	7.61 × 10^−06^	25	Cellular Assembly and Organization	1.72 × 10^−9^	Reproductive System Disease	1.31 × 10^−3^
Tissue morphology	7.61 × 10^−06^	56	Cellular Function and Maintenance	1.72 × 10^−9^	Cell Morphology	1.39 × 10^−3^
**Canonical pathways**	***p*** **value**	**Ratio**	Nutritional Disease	9.01 × 10^−8^	Nervous System Development and Function	2.80 × 10^−3^
Serotonin receptor signalling	5.77 × 10^−03^	4/46 (0.087)	Skeletal and Muscular System Development and Function	1.03 × 10^−7^	Cardiovascular System Development and Function	3.80 × 10^−3^
Xenobiotic metabolism signalling	2.73 × 10^−02^	12/294 (0.041)	Tissue Morphology	1.03 × 10^−7^	Protein Synthesis	5.38 × 10^−3^
LPS/IL-1 mediated inhibition of RXR function	3.18 × 10^−02^	10/235 (0.043)	Cell Morphology	2.63 × 10^−7^	Cellular Growth and Proliferation	6.00 × 10^−3^
Pentose phosphate pathway	3.60 × 10^−02^	3/80 (0.038)	Organismal Injury and Abnormalities	2.66 × 10^−7^	Auditory and Vestibular System Development and Function	6.15 × 10^−3^
cAMP-mediated signalling	3.80 × 10^−02^	10/219 (0.046)	Cellular Development	2.70 × 10^−7^	Behaviour	6.15 × 10^−3^

### Effects of all treatments in expression of specific genes and treatment-dependent changes in Galanin promoter methylation status

**Figure 6:**
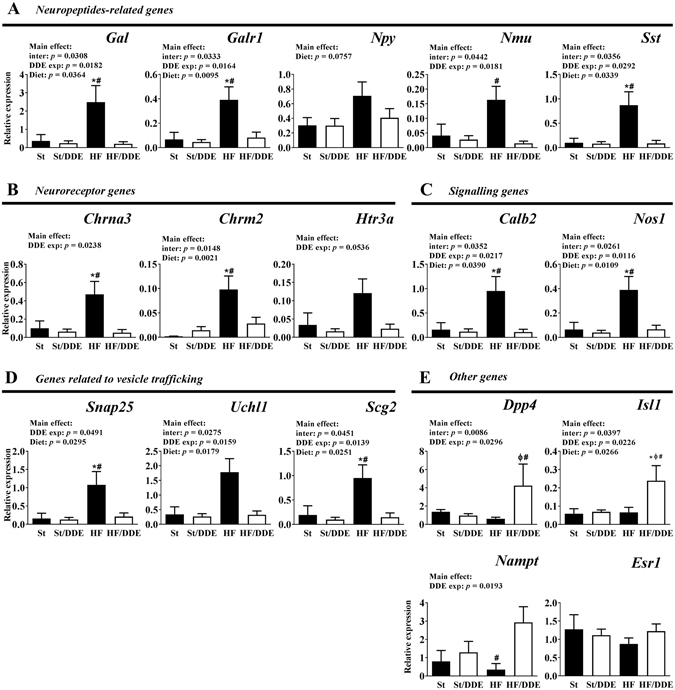
Gene expression analysis by quantitative real-time PCR (qRT-PCR) in visceral adipose tissue. Gene expression normalised to the housekeeping genes (*Hprt*, *Gusb* and *Tbp*). Statistical analysis with two-way ANOVA (main effects: diet, *p*,*p*’-DDE exposure and their interaction; *p* < 0.05), followed by Tukey’s multiple comparison post-hoc test: *p < 0.05 *vs* St, ^#^ *p* < 0.05 *vs* HF/DDE and ^Ø^ *p* < 0.05 *vs* St/DDE. St, standard diet; HF, high fat diet; *p*,*p*’-DDE, *p*,*p*’-dichlorodiphenyldichloroethylene.

**Figure 7:**
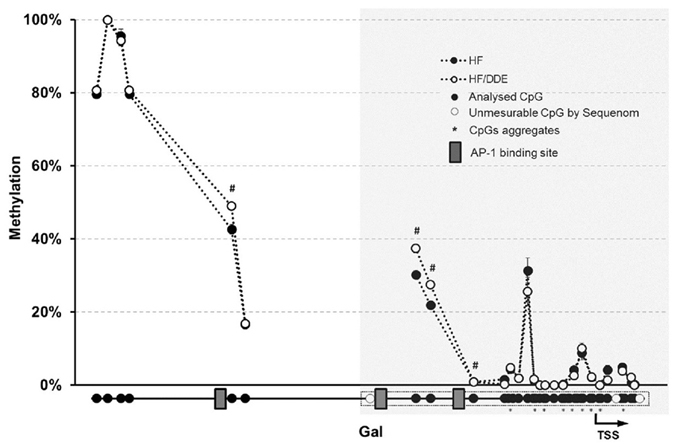
Methylation profile of galanin (*Gal*) promoter region in mesenteric vAT measured by Sequenom MassARRAY. The results are presented as average of percentage methylation ± SEM for each CpG site or aggregate of CpG sites. Statistical analysis unpaired *t* test: ^#^ *p* < 0.05. Gray area represents a CpG island. HF, high fat diet; *p*,*p*’-DDE, *p*,*p*’-dichlorodiphenyldichloroethylene; TSS, transcription start site.

Nervous system development and function-related genes were evaluated by qRT-PCR (Fig. 6). We observed an overall up-regulation of selected genes, namely neuropeptide-related genes *Gal*, *Npy*, *Nmu* and *Sst* and the galanin receptor 1 (*Galr1*), in HF group comparing to other treatments, including HF/DDE (significant interaction between HF diet and *p*,*p*’-DDE; exposure). Several neuroreceptor transcripts such as *Chrna3*, *Chrm2* (cholinergic receptor, muscarinic 2), and *Htr3a* (5-hydroxytryptamine receptor 3A), as well as calcium (calbindin 2; *Calb2*), nitric oxide (nitric oxide synthase 1, neuronal; *Nos1*) and vesicle trafficking-related genes followed the same pattern. In contrast, the concomitant exposure to HF diet and *p*,*p*’-DDE was associated with the up-regulation of *Dpp4*, *Isl1* and *Nampt* (nicotinamide phosphoribosyltransferase), this last with a *p*,*p*’-DDE-dependent increase. *Esr1* transcription did not show any difference between groups. From the nine genes with altered expression in HF/DDE animals that were selected for Sequenom analysis, only *Gal* (Fig. 7) showed changes in DNA methylation at its promoter region (see Supplementary Fig. S2). The HF animals (i.e. those with higher levels of expression) presented lower methylation of these CpGs comparing to HF/DDE and whilst the differences were small in magnitude and limited to a few CpGs sites, they were located near to AP-1 binding sites.

## Discussion

**Figure 8:**
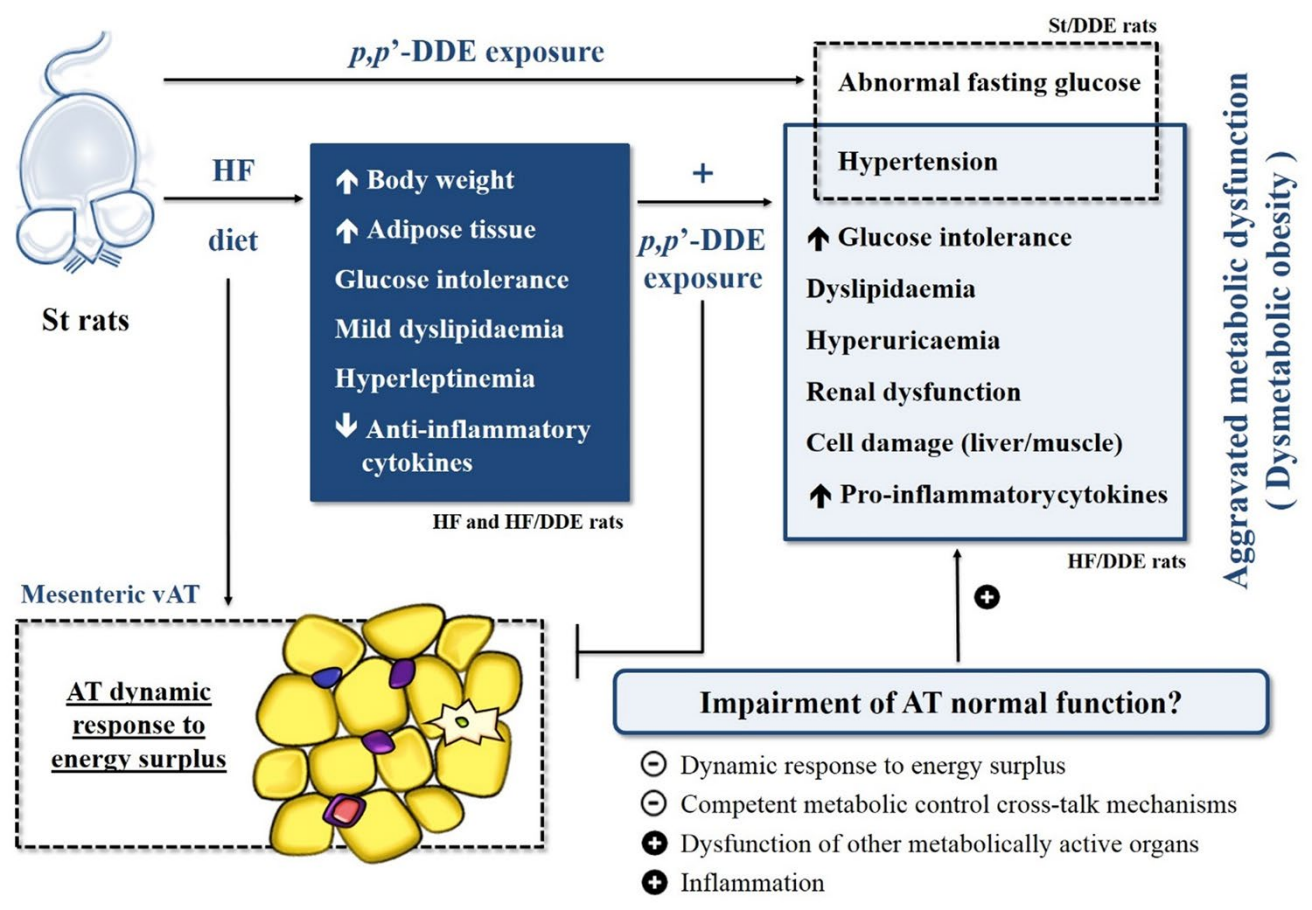
Schematic representation of the effects observed with the different treatments, whereby *p*,*p*’-DDE exposure appears to have an effect in metabolic syndrome evolution and dysmetabolic obesity, namely by exacerbating the effects of HF, in which the impairment of AT normal function seems to play a central role. St, standard diet; HF, high fat diet; *p*,*p*’-DDE, *p*,*p*’-dichlorodiphenyldichloroethylene; vAT, visceral adipose tissue.

The HF-fed rats exhibited several of the MetS features (Fig. 8) already described for this model^43, 44^, namely: a rapid weight gain, mostly due to a dramatic increase in AT mass; dysglycaemia with impaired response to glucose and tendentiously higher fasting insulin levels and HOMA index, mild dyslipidaemia; hyperleptinemia; and a reduction of the anti-inflammatory cytokine IL-10, alongside with a slight decrease in TGF-β1 levels, an immune system-modulating cytokine^45^.

However, our fundamental focus was on the assessment of *p*,*p*’-DDE exposure when supplied in an obesogenic context. Our study has revealed that HF-fed rats exposed to *p*,*p*’-DDE develop a severe and more exuberant metabolic dysfunction, independent of body weight changes (Fig. 8). The interaction between HF diet and *p*,*p*’-DDE exposure observed in this study corroborates the suggestion that contaminants in foods can contribute to the worsening of metabolic complications of obesity^25^, and allowed us to further explore their causal effects.

Accordingly, HF/DDE had a clear aggravation of glycaemic metabolism (Fig. 3A), with a significant effect of *p*,*p*’-DDE increasing fasting blood glucose levels and impairing glycaemia control with a pronounced tendency of increased fasting insulin levels and HOMA index for insulin resistance, effects to some extent already observed in St/DDE and in accordance with studies linking *p*,*p*’-DDE exposure to insulin resistance and T2D^8, 29, 40–42^.

In this context, the up-regulation of *Dpp4* transcription in HF/DDE mesenteric vAT (see Supplementary Table S2 and Fig. 6) could be contributing for their metabolic impairment worsening, as recently described^46, 47^. In fact, *Dpp4* (also known as CD26) is a ubiquitously expressed glycoprotein that is responsible for the rapid catalytic degradation of incretins, such as glucagon-like peptide-1 (GLP-1) and gastric inhibitory polypeptide (GIP), responsibles for an important part of postprandial insulin secretion. *DPP4* also mediates the degradation of many growth factors and hormones, chemokines and neuropeptides and is up regulated in pro-inflammatory states including obesity and T2D^48, 49^. Recently, *Dpp4* was also considered a novel cross-talk adipokine with autocrine and paracrine effects on insulin sensitivity impairment^48, 50^. On the other hand, insulin resistance may play an important role in the development of hypertension^51^. Corroborating epidemiological data^27, 52^, *p*,*p*’-DDE manifestly induced hypertension (Fig. 3B), an effect that was aggravated by the simultaneous ingestion of HF diet, but apparently distinct from those induced by HF diet alone.

Furthermore, one cannot overlook the probable effects of dyslipidaemia observed in HF/DDE animals (Fig. 3C), which, through lipotoxic events in insulin-responsive organs, may contribute to a reduction in peripheral insulin response. Indeed, *p*,*p*’-DDE exposure contributed to a more marked dyslipidaemia compared to HF diet alone, namely with higher HDL levels, the main transporter of cholesterol in rats. Additionally, without an overall increase in HF-fed rats NEFAs^53^, HF/DDE vAT lipolysis was increased (Fig. 2B), although in a smaller magnitude compared to St/DDE animals, probably due to an overwhelming effect of HF or a different *p*,*p*’-DDE concentration profile.

Taking into account an adipocentric view of metabolic dysfunction, we further evaluated the contribution of the mesenteric vAT impairment, considered determinant in the causal pathway of the MetS and dysmetabolic obesity^3, 4^. The AT inability to accommodate more lipids leads to functional and morphological changes, AT remodeling, in order to achieve a new equilibrium^39^. However, the apparent remodeling dynamics observed in HF group, characterized by higher proliferation and apoptotic indexes, was impaired by the interaction with *p*,*p*’-DDE exposure, patent in decreased proliferation and lesser percentage of bigger adipocytes, without an effect on mean adipocyte area (Fig. 5). Indeed, an expanded population of small and dysfunctional adipocytes, characterized by lower differentiation capacity and impaired capacity to control endocrine homeostasis, and therefore energy, metabolic and inflammatory homeostasis^24, 54, 55^, may contribute to obesity-associated insulin resistance^56^.

To further explore the underlying molecular mechanisms, we conducted a global gene expression evaluation between HF and HF/DDE, revealing a down-regulation of nervous system development and function-related genes, as well as of tissue development, signalling and metabolism-related genes (see Supplementary Table S2 and Table 1). To comprehend the importance of differences between HF groups, several genes of interest were evaluated in all treatments (Fig. 6). The higher expression of nervous system-related genes in HF rats compared to a non-obesogenic context, possibly in response to higher energy influx and AT increase as is known that high caloric intake leads to an increased sympathetic tone^57^, seem impaired when rats were also exposed to *p*,*p*’-DDE, possibly contributing to AT dysfunction. Indeed, the autonomic innervation of AT is important in the regulation of tissue mass and function through neuro-adipose junctions that also mediate leptin-driven lipolysis^58, 59^. Moreover, exposure to EDCs, namely *p*,*p*’-DDE, have been associated with several effects on the nervous system, acting not only as neurotoxicants but also disrupting neurotransmitter function^60, 61^.

These changes are demonstrative of the decrease in competent metabolic control cross-talk mechanisms, as AT is known to be innervated by the autonomic nervous system, especially by sympathetic nerves^62^. Moreover, parasympathetic innervation in AT, along with the ability of macrophages to participate in this cross-talk^63^, has been recently linked with cytokine production, inflammation, energy homeostasis and insulin resistance trough the cholinergic anti-inflammatory pathway^64^. Remarkably, several genes related to parasympathetic nervous system, such as cholinergic receptors and *Nos1*, were down-regulated.

Additionally, transcript levels of some neuropeptides followed the same pattern. *Gal*, the most altered gene (receptor also down-regulated), is involved in the positive regulation of food intake and the risk for obesity, but also contributes to the decrease of insulin resistance and blood pressure^65, 66^. Gal is engaged in the regulation of numerous physiological processes and distributed widely throughout the central and peripheral nervous system and other tissues, such as vAT. The mechanisms underlying the effects of Gal on appetite and obesity are its interaction with other appetite-regulating peptides, such as the positive interaction with Npy and negative with leptin, and the stimulation of muscle to use carbohydrates over lipids^67^. Indeed, as *in vivo* evidence suggest, Gal may counteract the metabolic disturbances induced by a HF diet, in part corroborated in this study, by means of the favouring of carbohydrate over lipid metabolism in muscle^67, 68^.

One can also hypothesize about the possible molecular mechanism involved in this HF diet x *p*,*p*’-DDE exposure interaction. As xenoestrogen, *p*,*p*’-DDE can interfere with estrogen signalling, namely in the induction of *Gal* ^69^. The considerable inhibition of *Esr1* transcription factor status in HF/DDE animals (see Supplementary Fig. S1), notwithstanding its unaltered transcription (Fig. 6), can be related with the *Isl1*-*ER* cross-talk ability to inhibit the ER-driven transcriptional activation^70^. Alternatively, compared to HF/DDE, the HF animals presented a decrease in DNA methylation at *Gal* regulatory region near to *AP-1* binding sites, which could also help explain its increased expression (Fig. 7). Once more, *p*,*p*’-DDE exposure demonstrated to counteract this effect.

Furthermore, the HF diet x *p*,*p*’-DDE exposure interaction led to an increase of inflammation (Fig. 4), with a striking increase in plasmatic IL-1β levels, to some extent also seen in St/DDE group, alongside a decline in anti-inflammatory cytokines. This cytokine, is implicated in T2D through the activation of the inflammasome, and important part of our innate immune system that responds to danger signals that are sensed by intracellular NOD-like receptors (NLRs), such as NLRP3 inflammasome that after activation facilitates caspase-1-dependent processing of pro-IL-1β into its active form^71^. Indeed, IL-1β is associated with visceral obesity and β cell failure^72, 73^, promotion of AT inflammation and limitation of fat expandability, ultimately contributing to ectopic lipid accumulation and disturbed fat-liver crosstalk^71, 73, 74^, as well as to cardiac arrhythmias^75^. In this regard, a negative correlation was observed between the weight of vAT and other metabolically active organs susceptible to accumulate lipids, especially liver (data not shown). Moreover, we already described that *p*,*p*’-DDE exposure associated with HF diet enhances the impairment of liver fatty acid composition and increases their overall levels, confirming a close relationship with hepatic lipid dysfunction^76^. Despite its well accepted relation with dysmetabolic obesity, the triggers that determine differential inflammasome activation remain to be identified^73^, emerging the environmental exposure to POPs, and EDCs in general, as another plausible mechanism.

This AT impairment contributes to the dysfunction of other metabolically active organs and interfere with its toxicological functions, namely of protection^13^. Indeed, HF/DDE animals presented a strong indication of cellular damage, with a significant increase of AST, AST/ALT and CK only in HF diet-fed animals exposed to *p*,*p*’-DDE, which may imply liver, kidney or muscle damage (see Supplementary Table S1). Ultimately, this damage can have a plausible relationship with the observed metabolic dysfunction, namely with glucose intolerance as a result of pancreas damage, expressed by an increase in total plasmatic amylase. Moreover, the observed renal dysfunction could also implicate this organ in the MetS aggravation by *p*,*p*’-DDE exposure^77^, expressed in higher glucose and urea excretion in HF/DDE animals, alongside with a dramatic increase of microalbuminuria. One can also speculate on the involvement of oxidative stress and hyperuricaemia, as well as the increase in lactate levels, on the development of insulin resistance and MetS^78^.

Taken together, our results confirm that *p*,*p*’-DDE exposure appears to have an important effect in MetS evolution and dysmetabolic obesity, exacerbating the effects of HF diet, in which the impairment of AT normal function seems to play an important role (Fig. 8). We verified that although *p*,*p*’-DDE exposure did not led to increased obesity, it contributed to a more exuberant MetS, aggravating some of its features, such as glucose intolerance, hypertension, dyslipidaemia and inflammation. In an adipocentric view of the genesis of MetS, the results of the mesenteric vAT evaluation also point to a deleterious effect of exposure to *p*,*p*’-DDE in a HF diet context. In addition, *p*,*p*’-DDE also exerts noteworthy independent effects, as the induction of hypertension, elevated fasting glucose and inflammation, even when not in an obesogenic context. The involvement of *p*,*p*’-DDE in these pathological developments emphasizes the need for a better understanding of EDCs mechanisms of action and their recognition as metabolism disrupting chemicals. To some extent, these results can help explain the interindividual variability of obesity effects and introduce these compounds as possible markers of dysmetabolic obesity.

## Methods

### Animal treatment

Twenty-four male Wistar rats, weighing 267 ± 11.8 g (8 weeks), were purchased from Charles River (Barcelona, Spain) and after at least 1 week of acclimatization under controlled environmental conditions (22–24 °C and 12 h light/dark cycles), were randomly divided into four groups and treated for 12 weeks: St, standard diet group; St/DDE, standard diet with *p*,*p*’-DDE exposed group; HF, high-fat diet group; HF/DDE, high-fat diet with *p*,*p*’-DDE exposed group. The *p*,*p*’-DDE exposure was applied in drinking water with the average concentration of 100 μg/kg/day (2.5 times less than lowest-observed-adverse-effect level). The “Standard” (Teklad 2014, Harlan Laboratories, Santiga, Spain) and “High Fat” (D1245 Research Diets, New Brunswick, USA) diets had, respectively, 13% and 45% of energy from lipids. Body weights were monitored weekly. For the extended treatment details see Supplementary Methods online. Animal handling and housing protocols followed European Union guidelines (Directive 2010/63/EU) and Portuguese Act (129/92) for the use of experimental animals. The protocol was approved by the Committee on the Ethics of Animal Experiments of the Faculty of Medicine of University of Porto.

### Evaluation of metabolic parameters

#### Oral glucose tolerance test (OGTT)

After 7 weeks of treatment, the rats were fasted for 4–6 hours and a baseline blood drawn from the saphenous vein was collected for plasma fasting glucose evaluation^79^. The rats were gavaged with a glucose solution (2 mg/g of body weight) and glycaemia measured thereafter until 120 min. Glucose levels were measured with Precision Xtra Plus test strips using a Optium Xceed device (Abbott Diabetes Care, Ltd., Maidenhead, UK). Plasma insulin levels at the end of the study were measured using a Rat/Mouse Insulin ELISA kit (Merck Milipore, Madrid, Spain). The homeostasis model assessment (HOMA) was used to calculate approximate insulin resistance using the formula: glucose (mg/dL) x insulin (ng/ml)/405^80^.

#### Systolic blood pressure evaluation

Measurement of SBP in conscious restrained rats was carried out by the noninvasive tail-cuff method^81^ using LE 5000 (Letica, Rochester Hills, USA) at weeks 4, 7 and 10. Each value of SBP was obtained by averaging out three to five consecutive similar measurements.

#### Blood and urine biochemical analysis

Biochemical evaluation of plasma and urine was performed at São João Hospital Center Clinical Pathology Department. Routine biochemical parameters were measured using conventional methods with an Olympus AU5400® automated clinical chemistry analyser (Beckman-Coulter®, Izasa, Porto, Portugal). Low-density lipoprotein (LDL) cholesterol was calculated according to Friedewald’s equation^82^. NEFAs were quantified using an *in vitro* enzymatic colorimetric method assay (Wako Chemicals GmbH, Neuss, Germany).

#### Cytokine profile evaluation

Interleukin-1 *beta* (IL-1β), IL-10), tumor necrosis factor-*alpha* (TNF-α) and transforming growth factor-*beta* 1 (TGF-β1) were measured with custom Milliplex Rat kits (Merck Millipore, Madrid, Spain), using Luminex xMAP Multiplexing Technology platform. Leptin, adiponectin and monocyte chemoattractant protein-1 (MCP-1) were determined using, respectively, Rat Leptin ELISA Kit (Merck Millipore, Madrid, Spain), Adiponectin Enzyme Immunoassay Kit and MCP-1 ELISA Kit (RayBiotech, Norcross, USA).

### Adipocyte isolation, lipolysis and determination of *p*,*p*’-DDE in the AT

Portions of visceral and subcutaneous AT (epididymal vAT and scAT, respectively) were used for adipocyte isolation according to the method of Rodbell *et al*.^83^ with some modifications, and determination of lipolysis (see Supplementary Methods online). The *p*,*p*’-DDE analysis was performed according to the method described by Fernandes & Pestana *et al*.^84^, and were expressed as µg of *p*,*p*’-DDE/kg of tissue or as *p*,*p*’-DDE burden in AT.

### AT morphology, apoptosis and proliferation

A portion of mesenteric vAT was fixed in buffered formaldehyde 10%, at least for 48 h (4 °C), dehydrated and finally embedded in paraffin. Three µm thick sections were obtained with a Leica® Microtome (RM2125RT, Lisbon, Portugal) for morphological analysis, apoptosis determination, and immunohistochemistry. Adipocyte size measurement was performed from hematoxylin-eosin–stained tissue sections as previously described^27^. Apoptosis was analysed using the terminal deoxynucleotidyl transferase-mediated deoxyuridine triphosphate nick end-labeling (Roche) and proliferation the immunohistochemical labeling of protein Ki67 protein^85^ (anti-Ki67, 1:50; fluorescein isothiocyanate-conjugated secondary antibody, 1:200; in 4% bovine serum albumin; Santa Cruz Biotechnology), with simultaneous of total DAPI-stained nuclei (4′,6-diamidine-2′-phenylindole dihydrochloride; Roche) count for the same optical fields. Apoptosis and proliferation were determined as the percentage of positive cells over total counted cells. Images from five randomly-selected different optical fields were acquired, under specimen identity occultation, with a Nikon Eclipse 50i^®^ microscope and analysed with ImageJ software^®^ (National Institute of Health, Bethesda, USA).

### AT total RNA isolation and microarrays

Total RNA was isolated from mesenteric vAT samples ground in liquid nitrogen, using RNA STAT-60 reagent (AMS Biotechnology, Abingdon, UK) followed by chloroform extraction and isopropanol precipitation. RNA extracts were treated with DNaseI to avoid contamination with genomic DNA and its concentration was assessed spectrophotometrically with a NanoDrop spectrophotometer (Thermo Scientific, Wilmington, DE, USA), and their integrity determined with the Agilent 2100 Bioanalyzer (Agilent Technologies, Massy, France). Only the high-quality RNA was processed and samples from HF and HF/DDE rats were used for the microarray analysis. Sample processing and data acquisition were carried out by the Genomics Core Lab of the University of Cambridge Biomedical Research Centre (Cambridge, UK). Biotinylated cRNA preparation and hybridization to Affymetrix Rat GeneChip^®^ Gene 1.0 ST Arrays were performed according to the recommended Affymetrix protocol (GeneChip Expression Analysis Technical Manual, Affymetrix, Santa Clara, CA, USA). Arrays were scanned and raw image data were converted to CEL files using Affymetrix Genechip Software.

### Microarray data analysis and functional profiling

All downstream analysis of microarray data was performed using Agilent’s GeneSpring GX 9 software (Agilent Technologies Inc. Santa Clara, USA). After importing the data, the CEL files were analysed under high stringency in order to reduce the number of false positives, as previously reported^86^. Two different analysis algorithms were used (robust multi-array average (RMA) and Plier analysis) and only genes whose expression patterns in each of the analyses were identical were taken forward for further study. Gene expression levels were considered significantly up- or down-regulated with a fold change of at least 1.25 fold with a *p* value of 5% (Student’s t-test). The pathway and biological analysis of gene expression data was performed using the Ingenuity pathway analysis software (IPA, Ingenuity^®^ Systems, Redwood City, USA).

### Gene expression analysis by quantitative real-time PCR (qRT-PCR)

To validate the microarray data, 10 differentially transcribed genes (up- and down-regulated) were selected for qRT-PCR analysis (see Supplementary Fig. S3). Several target genes’ expression levels were evaluated in all treatment groups. Total RNA (1 μg) was reverse transcribed to cDNA using Multiscribe Reverse Transcriptase with random primers according to the manufacturer’s protocol (Applied Biosystems). All cDNA samples were analysed in triplicate by qRT-PCRs conducted with SYBR green qPCR Mix (SYBR Green JumpStart Taq ReadyMix for qPCR, Sigma) on a 7500 Fast Real-Time PCR System (Applied Biosystems, UK). Gene expression was calculated using the 2^−ΔCT^ method^87^ and normalized against the geometric mean expression levels of the endogenous control genes: hypoxanthine-guanine-phosphoribosyltransferase (*Hprt*), β-glucuronidase (*Gusb*) and TATA box binding protein (*Tbp*). Gene-specific primers are listed in Supplementary Table S3.

### Sequenom MassARRAY quantitative methylation analysis

Genomic DNA was isolated from mesenteric vAT following standard methods and methylation analysis was performed following the protocol recommended by Sequenom (Sequenom, San Diego, USA). Aliquots of 0.5 μg were converted with sodium bisulphite using the EZ DNA Methylation™ Kit according to the manufacturer’s instructions (Zymo Research, Irvine, USA). Amplification of bisulphite-treated DNA (~2.5 ng μl^−1^) was performed using HotStarTaq DNA polymerase (Qiagen, UK) and primer pairs designed with Sequenom EpiDesigner (see parameters summarized in Supplementary Table S4). The PCR programme consisted of an initial 15 min denaturation at 94 °C followed by 45 cycles of 20 s at 94 °C, 30 s at the annealing temperature and 1 min at 72 °C. According to the manufacturer’s protocol (Sequenom Inc., CA, USA), the amplicons of bisulfite PCR were treated with shrimp alkaline phosphatase (SAP) followed by *in vitro* transcription with T7 RNA polymerase and base-specific cleavage using MassCLEAVE T cleavage kit. The samples were desalted and spotted on a 384-SpectroCHIP (Sequenom), followed by spectral acquisition on a MassARRAY Analyzer Compact MALDI-TOF MS (Sequenom). The resulting methylation calls were performed by the EpiTYPER software (Sequenom) to generate quantitative results for each CpG site or an aggregate of CpG sites. Three independent bisulphite-converted DNAs were analysed per sample. Quantification of methylation was performed in triplicate and the average of the two most concordant replicates was taken at each CpG site. The average methylation was calculated as a mean value of the CpG methylation values and expressed as percentage methylation.

### Statistical analysis

Values are expressed as the arithmetic mean ± standard error of the mean (SEM). Two-way ANOVA was used to determine the main effects of diet [Standard (St and St/DDE) vs High-fat diet (HF and HF/DDE)], *p*,*p*’-DDE exposure [non-exposed (St and HF) vs exposed Rats (St/DDE and HF/DDE)] and their interaction (diet x *p*,*p*’-DDE exposure). Posteriorly, tukey’s multiple comparison post-hoc test was used to determine differences between all experimental groups and represented with corresponding symbols. Two-way ANOVA repeated measures followed by Tukey’s multiple comparison post-hoc test was used to evaluate the differences between experimental conditions throughout time. To analyse the differences between 2 groups, a Student’s *t* test was used: unpaired *t* test when comparing methylation profile between treatment groups (HF and HF/DDE); regarding *p*,*p*’-DDE concentrations and *p*,*p*’-DDE burden in AT, unpaired *t* test when comparing between treatment groups (St/DDE and HF/DDE) and paired *t* test when comparing between different ATs from the same treatment group. The association between various parameters were analysed computing Pearson’s correlation coefficients. All statistical analyses were performed using GraphPad Prism 6 statistical software (GraphPad Software Inc., La Jolla, USA). The differences were considered statistically significant when *p* < 0.05.

## Electronic supplementary material

Supplementary Information
