# Supplementary material for: Adipose tissue dysfunction as a central mechanism leading to dysmetabolic obesity triggered by chronic exposure to p,p’-DDE
Source: Sci Rep. 2017 Jun 1;7:2738. doi: 10.1038/s41598-017-02885-9 (PMC5453948; doi:10.1038/s41598-017-02885-9)
Supplement: Supplementary file 1 — Supplementary Information [file 41598_2017_2885_MOESM1_ESM.pdf]

Supplementary Information

Adipose tissue dysfunction as a central mechanism leading to dysmetabolic obesity triggered by chronic exposure to *p,p*'-DDE

Diogo Pestana, Diana Teixeira, Manuela Meireles, Cláudia Marques, Sónia Norberto, Carla Sá, Virgínia C Fernandes, Luísa Correia-Sá, Ana Faria, Luísa Guardão, João T Guimarães, Wendy N Cooper, Ionel Sandovici, Valentina F Domingues, Cristina Delerue-Matos, Rosário Monteiro, Miguel Constância and Conceição Calhau

Table of Contents:

Figure S1. Top transcription factors associated with HF/DDE sensitive genes.......................Pg. 2

Figure S2. Methylation profile of *Dpp4*, *Mst4*, *Isl1*, *Gata5*, *Snap25*, *Uchl1*, *Crabp1* and *Npy* promoter regions in mesenteric vAT measured by Sequenom MassARRAY…......................Pg. 3

Figure S3. Validation of microarrays...Pg. 4

Table S1. Plasma and urine biochemical parameters …..………………..……........................Pg. 5

Table S2. Genes up- and down-regulated (expression microarray analysis)..........................Pg. 6

Table S3. Primers for gene expression analysis by quantitative real-time PCR…………....Pg. 21

Table S4. Summarized parameters of Sequenom primer pairs…..……..………...................Pg. 21

Supplementary methods.………………………………………….............................Pg. 22

Figure S1. Top transcription factors associated with HF/DDE sensitive genes. (A) Representative data network between transcription factors and modulated genes using IPA. Relative expression is depicted by green and red colouring to denote down- and up-regulation compared to HF animals, respectively.

Regulator	Regulation z-score	*p* value	Prediction activation state
**Up**			
HNF4A	**2.427**	9.70 x 10^{-2}	**activated**
STAT3	1.852	4.77 x 10^{-1}	
REST	1.815	2.84 x 10^{-11}	
ZNF217	1.382	1.23 x 10^{-2}	
HTT	0.950	8.48 x 10^{-6}	
**Down**			
ESR1	-1.730	3.58 x 10^{-1}	
PAX7	-1.181	4.07 x 10^{-2}	
POUAF1	-0.988	2.23 x 10^{-7}	
ISL1	-0.271	4.22 x 10^{-5}	

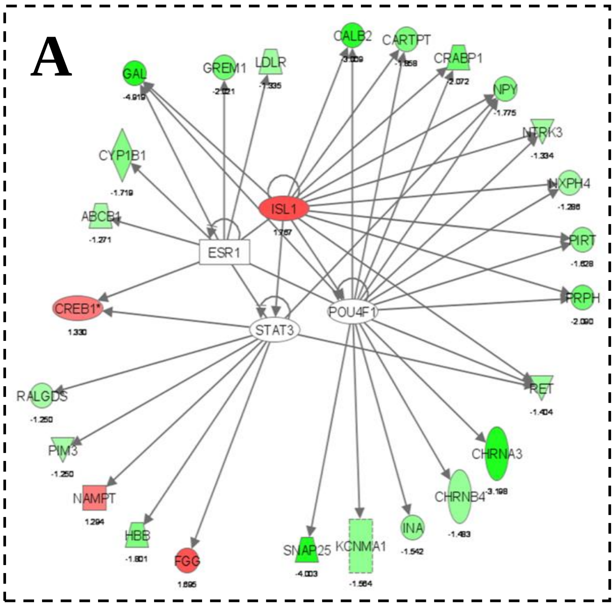

Figure S2. Methylation profile of *Dpp4*, *Mst4*, *Isl1*, *Gata5*, *Snap25*, *Uchl1*, *Crabp1* and *Npy* promoter regions in mesenteric vAT measured by Sequenom MassARRAY. The results are presented as average of percentage methylation ± SEM for each CpG site or aggregate of CpG sites. Statistical analysis unpaired *t* test: # $p < 0.05$. *p,p*'-DDE, *p,p*'-dichlorodiphenyldichloroethylene; HF, high fat diet; TSS, transcription start site.

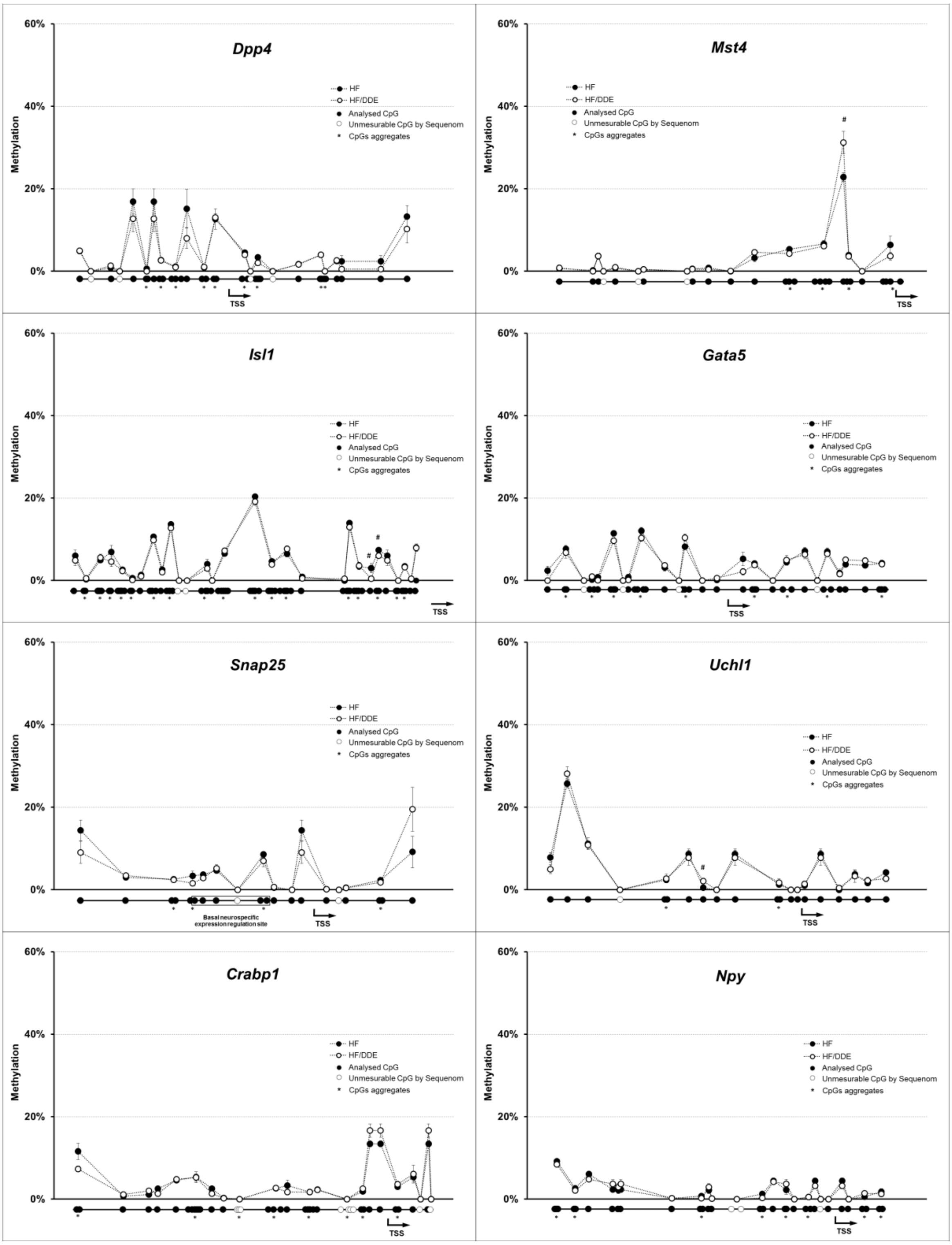

Figure S3. Validation of microarrays between HF/DDE and HF animals. Comparison of mRNA level of selected genes by microarray and qRT-PCR analysis. For the validation of microarray data, qRT-PCR results were analysed using REST 2009 ver. 2.0.13 (Qiagen, UK).

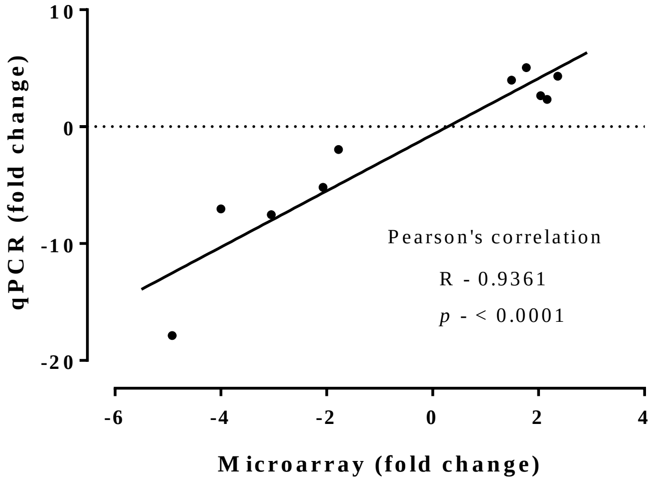

Table S1. Plasma and urine biochemical analyses performed at the end of treatment (12 weeks). Values are represented as mean ± SEM. Statistical analysis with two-way ANOVA (main effects: diet, *p,p*'-DDE exposure and their interaction; $p < 0.05$), followed by Tukey's multiple comparison post-hoc test: [a] $p < 0.05$ *vs* St group; [b] $p < 0.05$ *vs* St/DDE group; [c] $p < 0.05$ *vs* HF group. St, standard diet; HF, high fat diet; *p,p*'-DDE, *p,p*'-dichlorodiphenyldichloroethylene; AST, aspartate aminotransferase; ALT, alanine aminotransferase; ALP, alkaline phosphatise; CK, creatine kinase.

PLASMA	St	St/DEE	HF	HF/DDE	Main effect (*p* value)		
Parameters					Interaction	Diet	*p,p*'-DDE exposure
**Metabolic markers**							
Uric acid (mg/dL)	0.50 ± 0.07	0.43 ± 0.04	0.45 ± 0.05	0.78 ± 0.05 [abc]	**0.0015**	**0.0123**	**0.0238**
Lactate (mmol/L)	1.66 ± 0.15	1.46 ± 0.21	1.60 ± 0.12	2.35 ± 0.18 [abc]	**0.0114**	**0.0251**	n.s.
**Cell damage markers**							
Amylase (U/L)	517.33 ± 21.71	478.33 ± 12.96	528.17 ± 12.26	593.83 ± 27.92 [b]	**0.0156**	**0.0046**	n.s.
AST (U/L)	111.17 ± 22.14	83.33 ± 5.67	95.83 ± 5.96	157.83 ± 29.34 [ab]	**0.0484**	**0.0208**	n.s.
ALT (U/L)	33.00 ± 4.77	27.67 ± 1.69	26.17 ± 1.30	30.83 ± 3.06	n.s.	n.s.	n.s.
AST/ALT (U/L)	3.30 ± 0.19	3.06 ± 0.24	3.70 ± 0.27	5.02 ± 0.57 [ab]	**0.0376**	**0.0031**	n.s.
ALP (U/L)	86.50 ± 4.46	83.50 ± 7.21	94.00 ± 11.37	106.50 ± 11.62	n.s.	n.s.	n.s.
CK (U/L)	397.33 ± 123.42	300.50 ± 51.47	346.00 ± 56.64	758.50 ± 126.45 [abc]	**0.0241**	**0.0038**	**0.0134**
**URINE**	**St**	**St/DEE**	**HF**	**HF/DDE**	**Main effect (*p* value)**		
**Parameters**					**Interaction**	**Diet**	***p,p*'-DDE exposure**
**Metabolic markers**							
Glucose (mg/day)	1.56 ± 0.71	1.32 ± 0.47	2.56 ± 0.24	3.69 ± 1.15	n.s.	**0.0301**	n.s.
Microalbuminuria (mg/day)	0.04 ± 0.01	0.06 ± 0.01	0.05 ± 0.01	0.17 ± 0.06 [ac]	n.s.	**0.0473**	**0.0332**
Urea (mg/day)	561.54 ± 45.56	542.02 ± 49.91	662.47 ± 50.11	861.15 ± 200.69	n.s.	n.s.	n.s.
Creatinine (mg/day)	16.04 ± 0.56	16.46 ± 1.97	13.69 ± 0.68	18.91 ± 4.31	n.s.	n.s.	n.s.
**Ionogram**							
Sodium (mEq/day)	1.56 ± 0.10	1.62 ± 0.13	0.74 ± 0.08 [ab]	0.81 ± 0.16 [ab]	n.s.	**< 0.0001**	n.s.
Chlorine (mEq/day)	2.03 ± 0.08	2.14 ± 0.16	0.84 ± 0.08 [ab]	1.04 ± 0.28 [ab]	n.s.	**< 0.0001**	n.s.

Table S2. Down- and up- regulated genes following exposure to HF/DDE compared to HF.

Down-regulated genes

Gene	Fold change	p value	Affymetrix Cluster Id	Genebank	Description
**Gal**	**-4,92**	**0,01493**	10727321	J03624	galanin prepropeptide
**Snap25**	**-4,00**	**0,01634**	10840245	BC087699	synaptosomal-associated protein 25
**Chrna3**	**-3,20**	**0,01435**	10917597	X03440	cholinergic receptor, nicotinic, alpha 3
**Uchl1**	**-3,05**	**0,00925**	10776739	BC060573	ubiquitin carboxyl-terminal esterase L1 (ubiquitin thiolesterase)
**Syt1**	**-3,03**	**0,01105**	10902232	DQ181550	synaptotagmin I
**Calb2**	**-3,01**	**0,00829**	10810964	BC087603	calbindin 2
**Sst**	**-2,88**	**0,00795**	10751945	M25890	somatostatin
**Nmu**	**-2,75**	**0,01868**	10772231	M94555	neuromedin U
**Pcp4**	**-2,72**	**0,02419**	10750505	AJ493658	Purkinje cell protein 4
**Scg2**	**-2,69**	**0,01737**	10929263	M93669	secretogranin II (chromogranin C)
**Nefl**	**-2,63**	**0,01306**	10781266	AF031880	neurofilament, light polypeptide
**Rab3b**	**-2,45**	**0,01616**	10870847	BC060562	RAB3B, member RAS oncogene family
**Chrm2**	**-2,34**	**0,01555**	10854544	J03025	cholinergic receptor, muscarinic 2
**Stmn2**	**-2,19**	**0,00942**	10822358	BC087660	stathmin-like 2
**Prph**	**-2,09**	**0,01605**	10899061	AF031878	peripherin
**Ass1**	**-2,07**	**0,02046**	10835355	BC063146	argininosuccinate synthetase 1
**Crabp1**	**-2,07**	**0,01788**	10910089		cellular retinoic acid binding protein 1
**Tmem130**	**-2,04**	**0,00698**	10756597		transmembrane protein 130
**Grem1**	**-2,02**	**0,03137**	10848281	Y10019	gremlin 1, cysteine knot superfamily, homolog (Xenopus laevis)
**Ncam2**	**-1,98**	**0,00921**	10750002	AY495695	neural cell adhesion molecule 2
**Bcat1**	**-1,91**	**0,00507**	10866819	BC087710	branched chain aminotransferase 1, cytosolic
**Rab6b**	**-1,87**	**0,00605**	10912631		RAB6B, member RAS oncogene family
**Cartpt**	**-1,86**	**0,03882**	10820843	U10071	CART prepropeptide
**Znf804a**	**-1,85**	**0,00634**	10837241		zinc finger protein 804A
**Rasd1**	**-1,85**	**0,02462**	10743227	BC099136|AF239157	RAS, dexamethasone-induced 1
**Shc3**	**-1,83**	**0,00043**	10794261		SHC (Src homology 2 domain containing) transforming protein 3
**Stmn3**	**-1,83**	**0,01663**	10852531	AY004290	stathmin-like 3
**LOC287167**	**-1,82**	**0,02074**	10741765		globin, alpha
**Hbb**	**-1,80**	**0,02478**	10724315	M17084	hemoglobin, beta
**Slc7a10|Lrp3**	**-1,80**	**0,03128**	10706134	AB126813|BC127467|AB009463	solute carrier family 7, (neutral amino acid transporter, y+ system) member 10 | low density lipoprotein receptor-related protein 3
**L1cam**	**-1,79**	**0,00656**	10940195		L1 cell adhesion molecule
**Cpne4**	**-1,79**	**0,00701**	10912718		copine IV
**Disp2**	**-1,78**	**0,00483**	10838683		dispatched homolog 2 (Drosophila)
**Npy**	**-1,78**	**0,01635**	10855506	M15880	neuropeptide Y
	**-1,76**	**0,02189**	10939929		
**Elavl4**	**-1,74**	**0,02704**	10878712	BC158558	ELAV (embryonic lethal, abnormal vision, Drosophila)-like 4 (Hu antigen D)
**Cend1**	**-1,74**	**0,01097**	10726821	BC089963	cell cycle exit and neuronal differentiation 1
**Cyp1b1**	**-1,72**	**0,03501**	10887947	U09540	cytochrome P450, family 1, subfamily b, polypeptide 1

	**-1,72**	**0,01520**	10917486		
**Thy1**	**-1,72**	**0,00795**	10909407		Thy-1 cell surface antigen
**Dpp6**	**-1,72**	**0,01120**	10859886	M76426	dipeptidylpeptidase 6
**Scg3**	**-1,71**	**0,00541**	10918738	U02983	secretogranin III
**RGD1305733**	**-1,71**	**0,00868**	10731235	BC089943	similar to RIKEN cDNA 2900011O08
**Necab1**	**-1,70**	**0,02031**	10875616	AF193755	N-terminal EF-hand calcium binding protein 1
**Rab3c**	**-1,70**	**0,01626**	10821243	U54807	RAB3C, member RAS oncogene family
**Mab21l2**	**-1,69**	**0,04848**	10824089		mab-21-like 2 (C. elegans)
**Tpm2**	**-1,69**	**0,02920**	10876324	BC090009	tropomyosin 2, beta
**Lix1**	**-1,68**	**0,02193**	10703414		
**Syngr3**	**-1,67**	**0,00659**	10741203		synaptogyrin 3
**Camk2b**	**-1,67**	**0,00131**	10778268	M16112|AF069731	calcium/calmodulin-dependent protein kinase II beta
**Cadps**	**-1,67**	**0,00709**	10779390	U16802	Ca++-dependent secretion activator
**Syt5**	**-1,65**	**0,00424**	10704035	BC092198	synaptotagmin V
**Slc18a3**	**-1,65**	**0,01473**	10790340	U09211	solute carrier family 18 (vesicular acetylcholine), member 3
**Syp**	**-1,65**	**0,00817**	10937103	BC099798	synaptophysin
**Nos1**	**-1,64**	**0,01236**	10762455	U67309	nitric oxide synthase 1, neuronal
**RGD1565284**	**-1,63**	**0,00622**	10734472		similar to RIKEN cDNA A530088H08 gene
**Htr3a**	**-1,62**	**0,00761**	10917123		5-hydroxytryptamine (serotonin) receptor 3a
**Gabrb3**	**-1,61**	**0,01857**	10707582	AY742860	gamma-aminobutyric acid (GABA) A receptor, beta 3
**P2rx2**	**-1,60**	**0,00392**	10759383	AF020758|U14414|AF020759|AF020756|AF020757|Y10475|AF064549|L43511	purinergic receptor P2X, ligand-gated ion channel, 2
**Grp**	**-1,60**	**0,01160**	10802331	M31176	gastrin releasing peptide
**Galr1**	**-1,59**	**0,01118**	10805465	U30290	galanin receptor 1
**Pcsk1n**	**-1,59**	**0,01814**	10936976	AF181561	proprotein convertase subtilisin/kexin type 1 inhibitor
**Tac2**	**-1,59**	**0,03138**	10895980	M16410	tachykinin 2
**Ndrg4**	**-1,58**	**0,01138**	10809100	AY217032	N-myc downstream regulated gene 4
**Nrsn1**	**-1,58**	**0,02881**	10795077	BC166881	neurensin 1
	**-1,58**	**0,00014**	10860779		
**Vamp1**	**-1,57**	**0,01607**	10858858	BC092206	vesicle-associated membrane protein 1
**Kcnma1**	**-1,56**	**0,04434**	10778953	U40603|AF135265|AY344965|U55995|AB248959	potassium large conductance calcium-activated channel, subfamily M, alpha member 1
**Faim2**	**-1,56**	**0,01537**	10907165	BC087606	Fas apoptotic inhibitory molecule 2
**Ina**	**-1,54**	**0,02182**	10715900		internexin neuronal intermediate filament protein, alpha
**Mpped2**	**-1,54**	**0,00688**	10838312	AY093422	metallophosphoesterase domain containing 2
**Mapk10**	**-1,54**	**0,01520**	10771267	L27128	mitogen activated protein kinase 10
**Car14**	**-1,54**	**0,02398**	10825100	EF187253	carbonic anhydrase 14
**Slc7a14**	**-1,53**	**0,01123**	10814655		solute carrier family 7 (cationic amino acid transporter, y+ system), member 14
**Hspb6**	**-1,52**	**0,02774**	10706009	D29960	heat shock protein, alpha-crystallin-related, B6
**Sv2c**	**-1,52**	**0,02296**	10820613	AF060174	synaptic vesicle glycoprotein 2c
**Tmcc2**	**-1,51**	**0,03047**	10767597		transmembrane and coiled-coil domain family 2
**LOC500105**	**-1,51**	**0,00607**	10855138		similar to contactin associated protein-like 2 isoform a

**Gabra3**	**-1,51**	**0,00828**	10940090		gamma-aminobutyric acid (GABA) A receptor, alpha 3
**Aldoc**	**-1,50**	**0,01706**	10745095	BC099749	aldolase C, fructose-bisphosphate
**Ngfr**	**-1,50**	**0,00850**	10746538		nerve growth factor receptor (TNFR superfamily, member 16)
**Kcnb2**	**-1,50**	**0,01776**	10874952	M77482	potassium voltage gated channel, Shab-related subfamily, member 2
**Cd24**	**-1,50**	**0,02119**	10830624	BC064439	CD24 molecule
**Scgn**	**-1,50**	**0,00150**	10795162	AY513659	secretagogin, EF-hand calcium binding protein
**Adh1**	**-1,50**	**0,03483**	10819379	BC062403	alcohol dehydrogenase 1 (class I)
**Pclo**	**-1,50**	**0,00226**	10860421	AF227534|AF138789	piccolo (presynaptic cytomatrix protein)
**LOC681994**	**-1,49**	**0,01046**	10812390		similar to developmental endothelial locus-1 isoform b
**Htr1b**	**-1,49**	**0,00730**	10918979		5-hydroxytryptamine (serotonin) receptor 1B
**Syt17**	**-1,49**	**0,00278**	10725235	U30831	synaptotagmin XVII
**Lgi1**	**-1,48**	**0,00763**	10715146	BC089222	leucine-rich, glioma inactivated 1
**Chrnb4**	**-1,48**	**0,00861**	10917607	U42976	cholinergic receptor, nicotinic, beta 4
**Rit2**	**-1,48**	**0,04004**	10803571	BC091382	Ras-like without CAAX 2
**Mllt11**	**-1,48**	**0,00474**	10824965	BC087583	myeloid/lymphoid or mixed-lineage leukemia (trithorax homolog, Drosophila); translocated to, 11
**Dpp10**	**-1,48**	**0,03054**	10767203	DQ857325	dipeptidylpeptidase 10
**Kif1a**	**-1,47**	**0,00595**	10929937	AM180765	kinesin family member 1A
**Zcchc12**	**-1,47**	**0,01820**	10936360	BC089115	zinc finger, CCHC domain containing 12
	**-1,47**	**0,03937**	10770328		
**Gpr149**	**-1,47**	**0,03399**	10823412	AY030276	G protein-coupled receptor 149
**Cnr1**	**-1,47**	**0,00246**	10868186	U40395	cannabinoid receptor 1 (brain)
**Tubb2b**	**-1,46**	**0,03827**	10794824	BC105754	tubulin, beta 2b
**Resp18**	**-1,45**	**0,00273**	10929153	L25633	regulated endocrine-specific protein 18
**Nell2**	**-1,45**	**0,01221**	10906546	BC093617	NEL-like 2 (chicken)
**Snap91**	**-1,45**	**0,01742**	10919118		synaptosomal-associated protein 91
**Slc6a15**	**-1,45**	**0,03612**	10895251	L22022	solute carrier family 6 (neutral amino acid transporter), member 15
**Nell1**	**-1,44**	**0,02400**	10707275	U48246	NEL-like 1 (chicken)
**Tagln3**	**-1,43**	**0,01815**	10751048	AF459788	transgelin 3
	**-1,43**	**0,01977**	10808167		
	**-1,43**	**0,00537**	10759636		
**Gap43**	**-1,42**	**0,01072**	10751218	M16736	growth associated protein 43
**Rab3a**	**-1,42**	**0,00328**	10790912	BC087580	RAB3A, member RAS oncogene family
**Sntg2**	**-1,42**	**0,04256**	10889495		syntrophin, gamma 2
**Slc10a4**	**-1,42**	**0,01098**	10776582	AY704415	solute carrier family 10 (sodium/bile acid cotransporter family), member 4
**LOC689982|LOC316460|LOC689991**	**-1,42**	**0,00237**	10924035		similar to CG18437-PA
**S1pr3**	**-1,41**	**0,00184**	10797566		sphingosine-1-phosphate receptor 3
**Unc79**	**-1,41**	**0,00047**	10886509		unc-79 homolog (C. elegans)
**Sorcs1**	**-1,41**	**0,01065**	10730794		sortilin-related VPS10 domain containing receptor 1
**Tacr3**	**-1,41**	**0,02178**	10819139	J05189	tachykinin receptor 3
**Nsg2**	**-1,41**	**0,01873**	10741804	BC105916	neuron specific gene family member 2
**Tox**	**-1,41**	**0,00817**	10875363		thymocyte selection-associated high mobility group box
**Scube1**	**-1,41**	**0,00227**	10905843		signal peptide, CUB domain, EGF-like 1
**Ret**	**-1,40**	**0,02194**	10864918		ret proto-oncogene
**Ptprn**	**-1,40**	**0,00366**	10929128	U40652	protein tyrosine phosphatase, receptor type, N

**Crtac1**	**-1,40**	**0,01820**	10715431	U78304	cartilage acidic protein 1
**Ap3b2**	**-1,40**	**0,00242**	10723253		adaptor-related protein complex 3, beta 2 subunit
**Ank2**	**-1,40**	**0,01701**	10826616	U65916	ankyrin 2, neuronal
**LOC3060 96**	**-1,39**	**0,00377**	10785545		similar to Dachshund homolog 1 (Dach1)
**Map1a**	**-1,39**	**0,00319**	10839135	M83196	microtubule-associated protein 1A
**Entpd3**	**-1,39**	**0,02026**	10914354		ectonucleoside triphosphate diphosphohydrolase 3
**Phox2b**	**-1,39**	**0,03130**	10772629		paired-like homeobox 2b
**Phospho1**	**-1,39**	**0,00091**	10737659	BC169002	phosphatase, orphan 1
**Dgki**	**-1,39**	**0,02218**	10861843	AB058964	diacylglycerol kinase, iota
**Olfm1**	**-1,39**	**0,02987**	10834800	U03417	olfactomedin 1
**Reep1**	**-1,39**	**0,00514**	10856296		receptor accessory protein 1
**Wbp2**	**-1,39**	**0,01035**	10749108	AF499026	WW domain binding protein 2
**Wasf1**	**-1,39**	**0,01682**	10833952	BC091322	WAS protein family, member 1
**Unc5b**	**-1,38**	**0,01144**	10833013	U87306	unc-5 homolog B (C. elegans)
**Dpysl3**	**-1,38**	**0,02057**	10804245	AF389425	dihydropyrimidinase-like 3
**Atxn7l1**	**-1,38**	**0,00227**	10884189		ataxin 7-like 1
**Aldh1a1| Aldh1a7**	**-1,38**	**0,02809**	10714323	AF001898| M23995	aldehyde dehydrogenase 1 family, member A1 | aldehyde dehydrogenase family 1, subfamily A7
**Cnih2**	**-1,38**	**0,01831**	10727728	BC091325	cornichon homolog 2 (Drosophila)
**Mettl7b**	**-1,38**	**0,01381**	10899964	BC092587	methyltransferase like 7B
**Adcy1**	**-1,38**	**0,01161**	10774120		adenylate cyclase 1 (brain)
**Slc35d3**	**-1,37**	**0,01635**	10717053		solute carrier family 35, member D3
**Sgca**	**-1,37**	**0,02469**	10746458		sarcoglycan, alpha (dystrophin-associated glycoprotein)
**Dmpk**	**-1,37**	**0,02460**	10704728		dystrophia myotonica-protein kinase
**Slitrk1**	**-1,37**	**0,02513**	10785782		SLIT and NTRK-like family, member 1
**Wdr6|Dal rd3**	**-1,37**	**0,00508**	10920313	BC084708	WD repeat domain 6 | DALR anticodon binding domain containing 3
**Snurf|Snr pn**	**-1,37**	**0,03184**	10798943	AF101041| M29293	SNRPN upstream reading frame | small nuclear ribonucleoprotein polypeptide N
**Lrp12**	**-1,36**	**0,00559**	10903503		low density lipoprotein-related protein 12
**Cacna1e**	**-1,36**	**0,02219**	10768765	L15453	calcium channel, voltage-dependent, R type, alpha 1E subunit
	**-1,36**	**0,00480**	10854030		
**Tmem109**	**-1,36**	**0,01053**	10728812	BC078955	transmembrane protein 109
**Rab9b**	**-1,36**	**0,03220**	10939460		RAB9B, member RAS oncogene family
**Odz4**	**-1,36**	**0,01491**	10708695		odz, odd Oz/ten-m homolog 4 (Drosophila)
**Nmnat2**	**-1,36**	**0,02147**	10764736	DQ022370	nicotinamide nucleotide adenylyltransferase 2
**LOC6797 50**	**-1,36**	**0,01046**	10760568		similar to Salivary gland secretion 1 CG3047-PA
**Tcp11l1**	**-1,36**	**0,00271**	10847924		t-complex 11 like 1
**Clip3**	**-1,35**	**0,00804**	10705835		CAP-GLY domain containing linker protein 3
**Nkx2-3**	**-1,35**	**0,02542**	10730266		NK2 transcription factor related, locus 3 (Drosophila)
**RGD1562 449**	**-1,35**	**0,02558**	10867895		similar to hypothetical protein MGC2817
**Lrrn2**	**-1,35**	**0,00301**	10763933		leucine rich repeat neuronal 2
**Gpr81**	**-1,35**	**0,04268**	10758349	EU809461	G protein-coupled receptor 81
**Slc1a1**	**-1,35**	**0,00246**	10714616	D63772	solute carrier family 1 (neuronal/epithelial high affinity glutamate transporter, system Xag), member 1
**Pcdh1**	**-1,35**	**0,00557**	10804117	DQ863133	protocadherin 1
**Mylk**	**-1,35**	**0,04070**	10754510		myosin light chain kinase
**Cfl1**	**-1,34**	**0,01011**	10713074	BC086533	cofilin 1, non-muscle

**Tubb3**	**-1,34**	**0,04008**	10808702	BC097281	tubulin, beta 3
**Clstn3**	**-1,34**	**0,01286**	10865420		calsyntenin 3
**Gstm4|LOC100196943**	**-1,34**	**0,01786**	10825931	BC091199|FJ179403	glutathione S-transferase mu 4 | glutathione S-transferase mu 3
**Cntn5**	**-1,34**	**0,00993**	10914882	D87212	contactin 5
**Nkain1**	**-1,34**	**0,00702**	10880163		Na+/K+ transporting ATPase interacting 1
**LOC689963**	**-1,34**	**0,03625**	10859136		
**Tp53i11**	**-1,34**	**0,01158**	10838056	BC167758	tumor protein p53 inducible protein 11
**Ldlr**	**-1,34**	**0,00369**	10908521		low density lipoprotein receptor
**Ntrk3**	**-1,33**	**0,03820**	10722864	L14447|L14446|L03813|L14445|BC078844	neurotrophic tyrosine kinase, receptor, type 3
**Obfc2b**	**-1,33**	**0,00796**	10899775	BC104710	oligonucleotide/oligosaccharide-binding fold containing 2B
**Slc25a10**	**-1,33**	**0,01069**	10740159	BC081734	solute carrier family 25 (mitochondrial carrier; dicarboxylate transporter), member 10
**Gfra2**	**-1,33**	**0,01775**	10781410	AF003825	GDNF family receptor alpha 2
**Cacna1b**	**-1,33**	**0,00455**	10843229	AF055477	calcium channel, voltage-dependent, N type, alpha 1B subunit
**Hpca**	**-1,32**	**0,01335**	10879963	D12573	hippocalcin
**Sh2d3c**	**-1,32**	**0,01792**	10835604	BC158641	SH2 domain containing 3C
**G0s2**	**-1,32**	**0,00868**	10770807	BC088248	G0/G1switch 2
**Brunol4**	**-1,32**	**0,01466**	10803542		bruno-like 4, RNA binding protein (Drosophila)
**Bean**	**-1,32**	**0,01513**	10809044		brain expressed, associated with Nedd4
**MAST1**	**-1,32**	**0,00269**	10806601	AY227207	microtubule associated serine/threonine kinase 1
**Mapk8ip2**	**-1,32**	**0,01132**	10898606	BC105884|X57281	mitogen-activated protein kinase 8 interacting protein 2
**RGD1562533**	**-1,32**	**0,00492**	10760024	BC127538	similar to mKIAA0774 protein
**Fam70b**	**-1,32**	**0,02377**	10792734	BC169123	family with sequence similarity 70, member B
**Thbs4**	**-1,32**	**0,02993**	10820434	X89963	thrombospondin 4
**Astn1**	**-1,32**	**0,04297**	10764918		astrotactin 1
**Lfng**	**-1,32**	**0,00657**	10760594	BC070933	LFNG O-fucosylpeptide 3-beta-N-acetylglucosaminyltransferase
**Abcd1**	**-1,31**	**0,02778**	10935949		ATP-binding cassette, sub-family D (ALD), member 1
**Tgfb1i1**	**-1,31**	**0,02605**	10711364	AF314960	transforming growth factor beta 1 induced transcript 1
**Pnpla3**	**-1,31**	**0,01008**	10898203	BC161881	patatin-like phospholipase domain containing 3
**Kif3c**	**-1,31**	**0,00241**	10883311	AJ223599	kinesin family member 3C
**LOC685601**	**-1,31**	**0,00623**	10865236		similar to MICAL CG33208-PB, isoform B
**Ptprz1**	**-1,31**	**0,01869**	10853963	U09357	protein tyrosine phosphatase, receptor-type, Z polypeptide 1
**Dock6**	**-1,31**	**0,02326**	10908543	BC160867	dedicator of cytokinesis 6
	**-1,31**	**0,00970**	10884667		
**Rbpms2**	**-1,31**	**0,02106**	10911001	BC169038	RNA binding protein with multiple splicing 2
**RGD1307525|LOC292199**	**-1,31**	**0,01583**	10717421		similar to intracellular protein transport like (XM453) | hypothetical LOC292199
**Slc27a1**	**-1,31**	**0,03355**	10787313	BC074014	solute carrier family 27 (fatty acid transporter), member 1
**Maob**	**-1,30**	**0,01867**	10932211	M23601	monoamine oxidase B
**Olr1622**	**-1,30**	**0,00065**	10779788		olfactory receptor 1622
**Cby1**	**-1,30**	**0,00456**	10897698	AF393211	chibby homolog 1 (Drosophila)
**Lphn1**	**-1,30**	**0,00130**	10810236	AF081145|AF081147|U78105|AF081144|AF081146|U72487|	latrophilin 1

				AF111099	
**RGD1307 218**	**-1,30**	**0,00403**	10876730	BC089972	similar to RIKEN cDNA 2810432L12
**Smyd1**	**-1,30**	**0,01335**	10863104		SET and MYND domain containing 1
**Ap1s1**	**-1,30**	**0,00422**	10761042	BC169070	adaptor-related protein complex 1, sigma 1 subunit
**Tox3**	**-1,30**	**0,00120**	10806303	EU194254	TOX high mobility group box family member 3
**Nudt14**	**-1,30**	**0,01884**	10892381		nudix (nucleoside diphosphate linked moiety X)-type motif 14
**Gramd1b**	**-1,30**	**0,00037**	10916432		GRAM domain containing 1B
**Scube2**	**-1,30**	**0,04505**	10724792		signal peptide, CUB domain, EGF-like 2
**Dcun1d3**	**-1,30**	**0,00371**	10725340	BC097462	DCN1, defective in cullin neddylation 1, domain containing 3 (S. cerevisiae)
	**-1,29**	**0,00067**	10896630		
**Enah**	**-1,29**	**0,03434**	10770412	BC083927	enabled homolog (Drosophila)
**Gga1**	**-1,29**	**0,01165**	10897485	BC090031	golgi associated, gamma adaptin ear containing, ARF binding protein 1
**Tmem53**	**-1,29**	**0,00727**	10871419		transmembrane protein 53
**Dda1**	**-1,29**	**0,00044**	10787284		DET1 and DDB1 associated 1
**Dpysl5**	**-1,29**	**0,03159**	10889007	AB029432	dihydropyrimidinase-like 5
**Tmem90b**	**-1,29**	**0,01359**	10840613	BC092131	transmembrane protein 90B
	**-1,29**	**0,04738**	10818571		
	**-1,29**	**0,04751**	10908096		
**Dcun1d3**	**-1,29**	**0,00723**	10718643	BC097462	DCN1, defective in cullin neddylation 1, domain containing 3 (S. cerevisiae)
**Hspa2**	**-1,29**	**0,03935**	10885400	BC081803	heat shock protein alpha 2
**Repin1**	**-1,29**	**0,00147**	10855367	AY691175	replication initiator 1
**Slc6a8**	**-1,29**	**0,00337**	10935935	BC168238	solute carrier family 6 (neurotransmitter transporter, creatine), member 8
**Nxph4**	**-1,29**	**0,00790**	10903074	BC081805	neurexophilin 4
**Auts2l**	**-1,28**	**0,00686**	10761297		autism susceptibility candidate 2-like
**Synm**	**-1,28**	**0,03827**	10722694	AB091769	synemin, intermediate filament protein
**Zfp385a**	**-1,28**	**0,02102**	10907681	BC166729	zinc finger protein 385A
**Ttyh2**	**-1,28**	**0,00034**	10739399		tweety homolog 2 (Drosophila)
**Tmem177**	**-1,28**	**0,00613**	10767162	BC098688	transmembrane protein 177
**LOC6899 82|LOC31 6460|LOC 689991**	**-1,28**	**0,00185**	10924044		similar to CG18437-PA
**Prps1**	**-1,28**	**0,03272**	10935204	BC078853	phosphoribosyl pyrophosphate synthetase 1
	**-1,28**	**0,03200**	10871771		
**Zfpm2**	**-1,28**	**0,00109**	10896337		zinc finger protein, multitype 2
**Stxbp1**	**-1,28**	**0,00331**	10844390	BC088850	syntaxin binding protein 1
**Kcnk2**	**-1,28**	**0,02198**	10770637	AF325671	potassium channel, subfamily K, member 2
**Raver2**	**-1,28**	**0,03385**	10870240		ribonucleoprotein, PTB-binding 2
**Bcl9l**	**-1,28**	**0,00801**	10909527		B-cell CLL/lymphoma 9-like
**Arl2**	**-1,28**	**0,00436**	10728159		ADP-ribosylation factor-like 2
**Kcnb1**	**-1,28**	**0,00705**	10851943		potassium voltage gated channel, Shab-related subfamily, member 1
**Dnajc30**	**-1,27**	**0,04720**	10761225	BC166911	DnaJ (Hsp40) homolog, subfamily C, member 30
**Magmas**	**-1,27**	**0,03509**	10731783		mitochondria-associated protein involved in granulocyte-macrophage colony-stimulating factor signal transduction
**RGD1566 149**	**-1,27**	**0,00955**	10745222		similar to CDNA sequence BC017647
**Cnnm1**	**-1,27**	**0,00867**	10730272		cyclin M1
**Efemp2**	**-1,27**	**0,00992**	10713061	BC083804	EGF-containing fibulin-like extracellular matrix protein 2

**Abcb1a**	**-1,27**	**0,00800**	10853300	AF257746	ATP-binding cassette, sub-family B (MDR/TAP), member 1A
**Gabrg3**	**-1,27**	**0,04078**	10722328	M81142	gamma-aminobutyric acid (GABA) A receptor, gamma 3
**Anapc2**	**-1,27**	**0,00086**	10834168	BC166796	anaphase promoting complex subunit 2
**Alg3**	**-1,27**	**0,00654**	10752137	BC088475	asparagine-linked glycosylation 3, alpha-1,3- mannosyltransferase homolog (S. cerevisiae)
**Leng4**	**-1,27**	**0,03331**	10703715	BC169089	leukocyte receptor cluster (LRC) member 4
**Lrrn1**	**-1,27**	**0,03496**	10857541	BC107902	leucine rich repeat neuronal 1
**Ccdc69**	**-1,27**	**0,02666**	10742744		coiled-coil domain containing 69
**Chrna5**	**-1,27**	**0,01274**	10910133	J05231	cholinergic receptor, nicotinic, alpha 5
**Sardh**	**-1,27**	**0,00138**	10843938	AF067650	sarcosine dehydrogenase
**Myom1**	**-1,27**	**0,04698**	10925991	AY177416	myomesin 1
**Pdgfb**	**-1,27**	**0,03436**	10905521		platelet-derived growth factor beta polypeptide (simian sarcoma viral (v-sis) oncogene homolog)
	**-1,27**	**0,00265**	10723900		
**Niacr1**	**-1,27**	**0,01714**	10758351	AB103062	niacin receptor 1
**Chst2**	**-1,27**	**0,01549**	10919328		carbohydrate sulfotransferase 2
**Cox4i2**	**-1,26**	**0,02893**	10840895	AF255347	cytochrome c oxidase subunit IV isoform 2
**Brsk1**	**-1,26**	**0,00909**	10718881	AB365521	BR serine/threonine kinase 1
**Itga7**	**-1,26**	**0,01180**	10893267	BC088846|X65036|X74293	integrin, alpha 7
**Ldoc1l**	**-1,26**	**0,02076**	10905915		leucine zipper, down-regulated in cancer 1-like
**Arhgef18**	**-1,26**	**0,00134**	10759729		rho/rac guanine nucleotide exchange factor (GEF) 18
**Wiz**	**-1,26**	**0,00930**	10901002		widely-interspaced zinc finger motifs
**Sv2a**	**-1,26**	**0,03139**	10817512	L05435	synaptic vesicle glycoprotein 2a
**Gnao1**	**-1,26**	**0,02363**	10809428	M17526	guanine nucleotide binding protein (G protein), alpha activating activity polypeptide O
**Coro2b**	**-1,26**	**0,04235**	10918075	BC089991	coronin, actin binding protein, 2B
**Clip4**	**-1,26**	**0,02266**	10888620	BC081910	CAP-GLY domain containing linker protein family, member 4
**Atp6v0e2**	**-1,26**	**0,01621**	10855356	BC089958	ATPase, H+ transporting V0 subunit e2
**Syn1**	**-1,26**	**0,00226**	10932107	M27812	synapsin I
**Mccc2**	**-1,26**	**0,01067**	10820847	BC083581	methylcrotonoyl-Coenzyme A carboxylase 2 (beta)
**Ncam1**	**-1,26**	**0,01984**	10917183	BC101924	neural cell adhesion molecule 1
**H2afx**	**-1,26**	**0,00262**	10909480		H2A histone family, member X
**Syt15**	**-1,26**	**0,02256**	10786957	BC084685	synaptotagmin XV
	**-1,26**	**0,04970**	10887040		
**Praf2**	**-1,26**	**0,03522**	10937060		PRA1 domain family, member 2
**Brpf3**	**-1,26**	**0,00776**	10828791		bromodomain and PHD finger containing, 3
**Slc22a17|Efs**	**-1,25**	**0,01658**	10783669	AB040056|BC161942	solute carrier family 22, member 17 | embryonal Fyn-associated substrate
	**-1,25**	**0,03689**	10874048		
**Dhrs7b**	**-1,25**	**0,01134**	10734190	BC086453	dehydrogenase/reductase (SDR family) member 7B
**RGD1311249**	**-1,25**	**0,01454**	10876281	BC087107	similar to RIKEN cDNA B230312A22
**Gys1**	**-1,25**	**0,04707**	10706820	BC131849	glycogen synthase 1, muscle
	**-1,25**	**0,00187**	10842193	BC105769	
	**-1,25**	**0,00200**	10736415		
	**-1,25**	**0,01073**	10836249		
**Slc24a6**	**-1,25**	**0,00134**	10762304	BC079350	solute carrier family 24 (sodium/potassium/calcium exchanger), member 6
**Syt11**	**-1,25**	**0,04753**	10824349	AF000423	synaptotagmin XI
**Pom121**	**-1,25**	**0,00944**	10757674		nuclear pore membrane protein 121

**Chrnb2**	**-1,25**	**0,00941**	10824517	L31622	cholinergic receptor, nicotinic, beta 2 (neuronal)
**Slc7a4**	**-1,25**	**0,00555**	10752433	BC169079	solute carrier family 7 (cationic amino acid transporter, y+ system), member 4
**Ralgds**	**-1,25**	**0,02090**	10834817	L07925	ral guanine nucleotide dissociation stimulator
**Midn**	**-1,25**	**0,03337**	10900592		midnolin
**Pim3**	**-1,25**	**0,00922**	10898474	AF086624	pim-3 oncogene
**Pcbp4**	**-1,25**	**0,00678**	10912849		poly(rC) binding protein 4
**RGD1307966**	**-1,25**	**0,00781**	10747847		similar to hypothetical protein
**Pex6**	**-1,25**	**0,01633**	10921677	D63673	peroxisomal biogenesis factor 6
**Tbx2**	**-1,25**	**0,01995**	10737107		T-box 2
	**-1,25**	**0,02018**	10866408		
**Ergic3**	**-1,25**	**0,00595**	10841452		ERGIC and golgi 3
**Ndufb7**	**-1,25**	**0,03226**	10810299		NADH dehydrogenase (ubiquinone) 1 beta subcomplex, 7
**Prkce**	**-1,25**	**0,00909**	10882709	AY642593	protein kinase C, epsilon
**Syde1**	**-1,25**	**0,00457**	10894150		synapse defective 1, Rho GTPase, homolog 1 (C. elegans)
	**-1,25**	**0,00531**	10927051		
**Pcdh17**	**-1,25**	**0,01475**	10781745		protocadherin 17
**Srebf2**	**-1,25**	**0,00192**	10898091	BC101902	sterol regulatory element binding transcription factor 2
**Cbx4**	**-1,25**	**0,00047**	10931644		chromobox homolog 4 (Pc class homolog, Drosophila)
**Egfl7**	**-1,25**	**0,00008**	10834447	AF223678	EGF-like-domain, multiple 7

Up-regulated genes

Gene	Fold change	p value	Affymetrix Cluster Id	Genebank	Description
	**2,53**	**0,04082**	10915239		
**Dpp4**	**2,36**	**0,02772**	10845647	J04591	dipeptidylpeptidase 4
**Gpm6a**	**2,16**	**0,03081**	10791552	BC088862	glycoprotein m6a
**Mst4**	**2,04**	**0,00850**	10935418		serine/threonine protein kinase MST4
**Slc26a3**	**2,03**	**0,01657**	10884118	AF337809	solute carrier family 26, member 3
**Cmah**	**1,95**	**0,01695**	10798420	BC095843	cytidine monophosphate-N-acetylneuraminic acid hydroxylase
**Sbsn**	**1,92**	**0,04683**	10706059	BC107924	suprabasin
**LOC690930**	**1,90**	**0,01230**	10728930		similar to membrane-spanning 4-domains, subfamily A, member 6B
	**1,88**	**0,00304**	10877753	BC089062	
**Dsp**	**1,81**	**0,04772**	10797966		desmoplakin
**Upk1b|RGD1306995**	**1,80**	**0,04622**	10751239	BC093613	uroplakin 1B | similar to hypothetical protein FLJ32859
	**1,80**	**0,00014**	10934982		
**Isl1**	**1,77**	**0,02667**	10821486	AY557632	ISL LIM homeobox 1
	**1,74**	**0,01690**	10724580		
	**1,71**	**0,00229**	10797013		
**Pon3**	**1,71**	**0,02631**	10860878	BC079466	paraoxonase 3
**Scel**	**1,71**	**0,03495**	10781890	BC159429	sciellin
	**1,71**	**0,00234**	10932228		
	**1,70**	**0,04172**	10918833		
**Cxadr**	**1,70**	**0,02430**	10749983	BC088313	coxsackie virus and adenovirus receptor

**Fgg**	**1,69**	**0,03824**	10816067	BC078893	fibrinogen gamma chain
	**1,69**	**0,00015**	10772638		
**RGD1562 462**	**1,67**	**0,00016**	10770109		similar to Ifi204 protein
	**1,65**	**0,00156**	10802189		
**LOC3613 46**	**1,63**	**0,01180**	10805100	BC082051	similar to chromosome 18 open reading frame 54
	**1,61**	**0,00061**	10802541		
**RGD1563 835**	**1,60**	**0,00007**	10751237		similar to ribosomal protein L27
	**1,59**	**0,00129**	10858497		
	**1,58**	**0,00809**	10845070		
**Bcl6**	**1,58**	**0,00272**	10751931	BC166425	B-cell CLL/lymphoma 6
**Cyp2b2**	**1,58**	**0,01486**	10705230	M34452	cytochrome P450, family 2, subfamily b, polypeptide 2
	**1,58**	**0,01756**	10932310		
**Lrrc1**	**1,56**	**0,02945**	10918791	BC079423	leucine rich repeat containing 1
	**1,56**	**0,01173**	10924172		
	**1,56**	**0,03379**	10930588	K00160	
	**1,56**	**0,01288**	10838282		
	**1,55**	**0,00318**	10926095		
	**1,54**	**0,00022**	10813353		
**Lvrn**	**1,52**	**0,04120**	10801557		laeverin
**MGC108 823**	**1,52**	**0,02928**	10801975	BC089836	similar to interferon-inducible GTPase
**RGD1564 400**	**1,51**	**0,00003**	10836556		similar to Eukaryotic translation initiation factor 5 (eIF-5)
	**1,51**	**0,00059**	10936475		
**Samd9l**	**1,49**	**0,00559**	10860806		sterile alpha motif domain containing 9-like
**Samd9l**	**1,49**	**0,00564**	10860809		sterile alpha motif domain containing 9-like
**Samd9l**	**1,49**	**0,00565**	10860812		sterile alpha motif domain containing 9-like
	**1,49**	**0,00429**	10739223		
	**1,49**	**0,00164**	10744937		
**Gata5**	**1,49**	**0,02284**	10852378	BC085855	GATA binding protein 5
**LOC6833 02**	**1,47**	**0,00002**	10787048		similar to tumor protein, translationally-controlled 1
	**1,47**	**0,00015**	10897891		
	**1,47**	**0,02787**	10708589		
	**1,47**	**0,00057**	10730472		
**RGD1564 552**	**1,47**	**0,00069**	10799888		similar to ribosomal protein L21
	**1,46**	**0,00055**	10728028	BC090353	
**Ddx3x**	**1,46**	**0,00082**	10936753	BC085914	DEAD (Asp-Glu-Ala-Asp) box polypeptide 3, X-linked
**LOC5006 25**	**1,45**	**0,00221**	10883443		
	**1,45**	**0,00059**	10905770		
**Samd9l**	**1,45**	**0,00637**	10860815	BC098747	sterile alpha motif domain containing 9-like
**Cir1**	**1,44**	**0,00001**	10796921	BC085869	corepressor interacting with RBPJ, 1
	**1,44**	**0,00007**	10868302	AY539927	
	**1,44**	**0,00125**	10715250		
**Gal3st2**	**1,44**	**0,03251**	10925678		galactose-3-O-sulfotransferase 2
	**1,43**	**0,00028**	10853149		
	**1,43**	**0,00066**	10721218		

	**1,43**	**0,00002**	10843207		
	**1,43**	**0,00041**	10853681		
	**1,42**	**0,03298**	10802710		
	**1,42**	**0,00249**	10772861		
**Rgs2**	**1,42**	**0,00766**	10768332	AY043246	regulator of G-protein signaling 2
	**1,41**	**0,03643**	10749975		
**Herc6**	**1,41**	**0,00275**	10862765	BC085921	hect domain and RLD 6
	**1,41**	**0,00002**	10911925		
**Egln1**	**1,41**	**0,00162**	10724578	AY228140	EGL nine homolog 1 (C. elegans)
	**1,40**	**0,00226**	10761253		
	**1,40**	**0,00156**	10903522		
	**1,40**	**0,00173**	10895391		
	**1,40**	**0,02737**	10859195		
**Tbc1d22a |RGD1560617**	**1,39**	**0,00150**	10898444	AF062594	TBC1 domain family, member 22a | hypothetical gene supported by NM_053561; AF062594
	**1,39**	**0,00080**	10906590		
	**1,39**	**0,00651**	10910770		
	**1,39**	**0,00089**	10742962		
	**1,39**	**0,00525**	10852671		
	**1,39**	**0,00959**	10796230		
**LOC681338**	**1,39**	**0,00119**	10928032		similar to ribosomal protein L31
	**1,39**	**0,00154**	10785473		
**Spock2**	**1,39**	**0,01691**	10829965		sparc/osteonectin, cwcv and kazal-like domains proteoglycan 2
**Cobl**	**1,38**	**0,03544**	10778525		cordon-bleu homolog (mouse)
	**1,38**	**0,00123**	10918865		
	**1,38**	**0,00019**	10926819		
	**1,38**	**0,00302**	10813885		
	**1,38**	**0,00712**	10768303		
	**1,37**	**0,00142**	10792331		
	**1,37**	**0,01419**	10765036		
	**1,37**	**0,00431**	10859117		
**RGD1561086**	**1,37**	**0,00085**	10934113		similar to ribosomal protein L34
**LOC100361110**	**1,36**	**0,00615**	10917992		histone H3.3B-like
**Nphs1**	**1,36**	**0,01611**	10705892	AF161715	nephrosis 1 homolog (human)
	**1,36**	**0,02866**	10751789		
	**1,36**	**0,00295**	10810278		
	**1,36**	**0,00133**	10836015		
	**1,36**	**0,01071**	10723464		
	**1,36**	**0,02950**	10772272		
	**1,36**	**0,03501**	10767044		
**Ezr**	**1,36**	**0,02138**	10717891	BC081958	ezrin
**LOC304239**	**1,36**	**0,04208**	10756334	BC166500	similar to RalA binding protein 1
**RGD1565170**	**1,36**	**0,00002**	10780763		similar to 60S ribosomal protein L23a
**C1galt1**	**1,36**	**0,00279**	10853691	AF157963	core 1 synthase, glycoprotein-N-acetylgalactosamine 3-beta-galactosyltransferase, 1

**Tlr7**	**1,35**	**0,02577**	10933345	EF032637	toll-like receptor 7
	**1,35**	**0,00026**	10890535	M19635	
**LOC684806**	**1,35**	**0,00052**	10781564		similar to 40S ribosomal protein S29
**Giot1|Zfp347**	**1,35**	**0,00068**	10900841	AB047636|AB047637	gonadotropin inducible ovarian transcription factor 1 | zinc finger protein 347
	**1,35**	**0,00128**	10890297		
	**1,35**	**0,00214**	10889421		
	**1,34**	**0,03294**	10807793		
	**1,34**	**0,00715**	10754114		
	**1,34**	**0,00307**	10838352		
	**1,34**	**0,00489**	10755670		
	**1,34**	**0,00011**	10768301		
	**1,34**	**0,00381**	10785895		
	**1,33**	**0,01627**	10713604		
	**1,33**	**0,02376**	10930569	BC166549	
	**1,33**	**0,00041**	10918653		
**RGD1561736**	**1,33**	**0,00162**	10836017		similar to ribosomal protein L10
**Cnot7**	**1,33**	**0,00280**	10787209	BC167766	CCR4-NOT transcription complex, subunit 7
**Gtf2a2**	**1,33**	**0,00416**	10911309	BC168727	general transcription factor IIA, 2
**Tp53inp1**	**1,33**	**0,00358**	10867609	AB107917	tumor protein p53 inducible nuclear protein 1
**Tbpl1**	**1,33**	**0,00204**	10702309		TATA box binding protein-like 1
	**1,33**	**0,00106**	10803671		
**RGD1310507**	**1,33**	**0,03873**	10919658		similar to RIKEN cDNA 1300017J02
**Rhpn2**	**1,33**	**0,04850**	10706146		rhophilin, Rho GTPase binding protein 2
	**1,33**	**0,00609**	10900122		
	**1,33**	**0,00098**	10923938		
**LOC100362277**	**1,33**	**0,00051**	10808893		60S ribosomal protein L29-like
**RGD1560471**	**1,33**	**0,00012**	10804693	BC168236	similar to hypothetical protein 4933429F08
**Chordc1**	**1,33**	**0,00018**	10908133		cysteine and histidine-rich domain (CHORD)-containing 1
	**1,32**	**0,00008**	10802381		
**MGC109340**	**1,32**	**0,02350**	10935031	BC092634	similar to Microsomal signal peptidase 23 kDa subunit (SPase 22 kDa subunit) (SPC22/23)
	**1,32**	**0,00175**	10859627		
	**1,32**	**0,03333**	10926089		
	**1,32**	**0,00004**	10731222		
	**1,32**	**0,00687**	10840657		
	**1,32**	**0,00054**	10813246		
**Ube2v2**	**1,32**	**0,00230**	10752621	BC087593	ubiquitin-conjugating enzyme E2 variant 2
	**1,32**	**0,00002**	10752061		
	**1,32**	**0,01579**	10877667		
	**1,32**	**0,00185**	10787722		
	**1,32**	**0,00215**	10787782	BC099122	
	**1,32**	**0,02256**	10870488		
	**1,31**	**0,00101**	10739167		
**Mospd1**	**1,31**	**0,00331**	10939816	BC086521	motile sperm domain containing 1
**Cgn**	**1,31**	**0,01715**	10824860		cingulin

	**1,31**	**0,00216**	10863570		
**Sugt1**	**1,31**	**0,00353**	10781699	BC158724	SGT1, suppressor of G2 allele of SKP1 (S. cerevisiae)
	**1,31**	**0,01642**	10774625		
	**1,31**	**0,03036**	10824788		
**Clk4**	**1,31**	**0,00424**	10733321	BC079006	CDC like kinase 4
**Orc4l**	**1,31**	**0,00587**	10845095	BC060516	origin recognition complex, subunit 4-like (yeast)
	**1,31**	**0,00417**	10930564		
**Mospd1**	**1,31**	**0,00320**	10778399	BC086521	motile sperm domain containing 1
**Tmsb4x**	**1,31**	**0,00079**	10876291	M34043	thymosin beta 4, X-linked
**Znf654**	**1,31**	**0,00513**	10752654		zinc finger protein 654
	**1,30**	**0,01466**	10933038		
**LOC679469**	**1,30**	**0,00119**	10786359		similar to retinoblastoma-associated protein 140
	**1,30**	**0,00180**	10860325		
**Tc2n**	**1,30**	**0,01840**	10891765	BC088432	tandem C2 domains, nuclear
**RGD1561736**	**1,30**	**0,00375**	10940411		similar to ribosomal protein L10
	**1,30**	**0,02798**	10804402		
**Morf4l2**	**1,30**	**0,00175**	10939444	BC083606	mortality factor 4 like 2
**N5**	**1,30**	**0,00834**	10861560	L31882	
**RGD1565054**	**1,30**	**0,00040**	10938483		similar to 60S acidic ribosomal protein P1
	**1,30**	**0,00007**	10741861		
**Set|Pkn3**	**1,30**	**0,00128**	10749869	BC158573	SET nuclear oncogene | protein kinase N3
**Adamts19**	**1,30**	**0,00015**	10801960		ADAM metallopeptidase with thrombospondin type 1 motif, 19
**Fam96a**	**1,30**	**0,01556**	10911042	BC086524	family with sequence similarity 96, member A
	**1,30**	**0,00407**	10718421		
**Ifi204**	**1,30**	**0,01471**	10770082	BC085891	interferon activated gene 204
	**1,30**	**0,00281**	10860184		
	**1,29**	**0,00072**	10899428	BC063811	
**Sult1d1**	**1,29**	**0,03365**	10771919	U32372	sulfotransferase family 1D, member 1
	**1,29**	**0,00049**	10804560		
**Nampt**	**1,29**	**0,02055**	10884162	AB081730	nicotinamide phosphoribosyltransferase
	**1,29**	**0,01599**	10742386		
**RGD1566035**	**1,29**	**0,00014**	10936278		similar to protein tyrosine phosphatase 4a1
**Erp44**	**1,29**	**0,00093**	10876675	AY158662	endoplasmic reticulum protein 44
	**1,29**	**0,00340**	10884046		
	**1,29**	**0,00491**	10726672		
**Ttc35**	**1,29**	**0,00000**	10896380	BC158805	tetratricopeptide repeat domain 35
**H3f3b**	**1,29**	**0,02752**	10749070	BC063159	H3 histone, family 3B
**Sf3b1**	**1,29**	**0,00281**	10928122		splicing factor 3b, subunit 1
	**1,29**	**0,01620**	10852392		
	**1,29**	**0,00232**	10800832		
**Npm1**	**1,29**	**0,00650**	10741907	J04943	nucleophosmin (nucleolar phosphoprotein B23, numatrin)
**RGD1304929**	**1,29**	**0,00226**	10781482		similar to chromosome 13 open reading frame 18
**Tiprl**	**1,29**	**0,00005**	10769509	BC128780	TIP41, TOR signaling pathway regulator-like (S. cerevisiae)
**Slc28a2**	**1,29**	**0,00650**	10849279	AY029302	solute carrier family 28 (sodium-coupled nucleoside transporter), member 2

**Ntn1|RGD1564148**	**1,29**	**0,03096**	10743781		netrin 1 | similar to microfibrillar-associated protein 1
	**1,29**	**0,00405**	10760068	BC091375	
**RGD1562952**	**1,28**	**0,00087**	10812779		similar to Erbb2 interacting protein isoform 2
**LOC292449**	**1,28**	**0,01029**	10701663	AY389467	similar to hypothetical protein
	**1,28**	**0,00629**	10743855		
	**1,28**	**0,00248**	10772758		
	**1,28**	**0,00064**	10905605		
**Tomm70a**	**1,28**	**0,00071**	10884211	BC098640	translocase of outer mitochondrial membrane 70 homolog A (S. cerevisiae)
	**1,28**	**0,00041**	10801535		
	**1,28**	**0,00405**	10784219		
**Cul4b**	**1,28**	**0,00340**	10931893		cullin 4B
	**1,28**	**0,01807**	10881474	BC168232	
**Rex2**	**1,28**	**0,00281**	10873880	BC168232	reduced expression 2
	**1,28**	**0,01021**	10826604		
**Matr3**	**1,28**	**0,01313**	10781787	AB205483	matrin 3
	**1,28**	**0,00960**	10775624		
	**1,28**	**0,04548**	10845645		
**Krt7**	**1,28**	**0,03367**	10899405		keratin 7
	**1,28**	**0,00050**	10807872		
	**1,28**	**0,00604**	10727600		
**RGD1564843**	**1,28**	**0,00005**	10875815		similar to hypothetical protein 4930474N05
**Psmc6**	**1,28**	**0,00179**	10779607		proteasome (prosome, macropain) 26S subunit, ATPase, 6
**LOC690662**	**1,28**	**0,00159**	10808010		similar to 60S ribosomal protein L29 (P23)
**Eri1**	**1,28**	**0,00358**	10788497	BC089828	exoribonuclease 1
**RGD1309095**	**1,28**	**0,01100**	10927712		similar to hypothetical protein BC015148
	**1,28**	**0,00003**	10702187		
	**1,28**	**0,00011**	10751799		
**Clic2**	**1,27**	**0,00029**	10827592	BC088182	chloride intracellular channel 2
**Sp1**	**1,27**	**0,00338**	10899552	AY305388	
	**1,27**	**0,00315**	10791820		
	**1,27**	**0,01336**	10793838		
	**1,27**	**0,03050**	10804301		
**Enc1**	**1,27**	**0,02941**	10812689	AY669396	ectodermal-neural cortex 1
	**1,27**	**0,01347**	10812881		
**Rpe**	**1,27**	**0,00157**	10924076	BC101869	ribulose-5-phosphate-3-epimerase
**RGD735140**	**1,27**	**0,00506**	10850140		hypothetical protein LK44
	**1,27**	**0,00766**	10850440		
**LOC690096**	**1,27**	**0,00406**	10774432		similar to ribosomal protein L28
	**1,27**	**0,01781**	10909614		
**Shroom2**	**1,27**	**0,01424**	10937601	BC085701	shroom family member 2
	**1,27**	**0,00650**	10867361		
	**1,27**	**0,03290**	10714830		
**RGD1563157**	**1,27**	**0,00001**	10883799		similar to 60S ribosomal protein L35

**LOC680027**	**1,27**	**0,00212**	10882523		similar to suppressor of initiator codon mutations, related sequence 1
**Eif3e**	**1,27**	**0,01516**	10903545	BC082087	eukaryotic translation initiation factor 3, subunit E
**LOC686066**	**1,27**	**0,00114**	10844181		similar to 60S ribosomal protein L38
	**1,27**	**0,01247**	10811008		
	**1,27**	**0,00025**	10877067		
	**1,26**	**0,01000**	10903674		
**RGD1311863**	**1,26**	**0,01358**	10714264	BC091268	similar to RIKEN cDNA 2410127L17
**tGap1|LOC304239|LOC688241**	**1,26**	**0,00128**	10843142	AY631396|BC166500	GTPase activating protein testicular GAP1 | similar to RalA binding protein 1 | similar to GTPase activating protein testicular GAP1
	**1,26**	**0,03567**	10840653	BC166814	
**Stag2**	**1,26**	**0,00597**	10936163		stromal antigen 2
	**1,26**	**0,01605**	10737647		
**Tprkb**	**1,26**	**0,00280**	10856914	BC087060	Tp53rk binding protein
	**1,26**	**0,00013**	10830972		
**Il22ra1**	**1,26**	**0,03193**	10872876		interleukin 22 receptor, alpha 1
	**1,26**	**0,01872**	10710152		
**Spopl**	**1,26**	**0,01729**	10843172	BC167106	speckle-type POZ protein-like
	**1,26**	**0,01109**	10788345		
**Zbp1**	**1,26**	**0,01752**	10852136	AJ302054	Z-DNA binding protein 1
**Ndufb4-ps1**	**1,26**	**0,02476**	10906544		NADH dehydrogenase (ubiquinone) 1 beta subcomplex 4, pseudogene 1
	**1,26**	**0,00313**	10769693		
	**1,26**	**0,00318**	10772726		
	**1,26**	**0,00019**	10818134		
**Dcun1d5**	**1,26**	**0,02981**	10907849	BC087627	DCN1, defective in cullin neddylation 1, domain containing 5 (S. cerevisiae)
**Slc25a32**	**1,26**	**0,02340**	10903482	BC166530	solute carrier family 25, member 32
**Yme1l1**	**1,26**	**0,00131**	10796800	BC081751	YME1-like 1 (S. cerevisiae)
	**1,26**	**0,00485**	10850519		
**Gnl3**	**1,26**	**0,00665**	10790002	BC093602	guanine nucleotide binding protein-like 3 (nucleolar)
	**1,26**	**0,00302**	10939178		
**Tpd52l3**	**1,26**	**0,01880**	10714754		tumor protein D52-like 3
**Hmgb1|Hmg1l1|RGD1562312|LOC678705**	**1,26**	**0,00295**	10763430	AF275734|BC168143	high mobility group box 1 | high-mobility group (nonhistone chromosomal) protein 1-like 1 | similar to High mobility group protein 1 (HMG-1) | hypothetical protein LOC678705
	**1,26**	**0,03708**	10896964		
**Snx6**	**1,26**	**0,00378**	10889944	BC168856	sorting nexin 6
	**1,26**	**0,02735**	10778080		
	**1,26**	**0,00079**	10940003		
	**1,26**	**0,00530**	10714053		
**Paip2l1**	**1,25**	**0,00027**	10710769	BC079261	polyadenylate-binding protein-interacting protein 2-like 1
	**1,25**	**0,00376**	10881468	BC168232	
**Psma3|Psma3l**	**1,25**	**0,00638**	10885045	BC081817|M58593	proteasome (prosome, macropain) subunit, alpha type 3 | proteasome subunit alpha type 3-like
**LOC498750**	**1,25**	**0,00253**	10798386	BC089212|BC085940	similar to cDNA sequence BC005537
**Trnt1**	**1,25**	**0,00984**	10857538	BC095841	tRNA nucleotidyl transferase, CCA-adding, 1

	**1,25**	**0,00863**	10927699		
**Mphosph 10**	**1,25**	**0,03530**	10722566	BC161909	M-phase phosphoprotein 10 (U3 small nucleolar ribonucleoprotein)
	**1,25**	**0,00157**	10851813		
**Son**	**1,25**	**0,01993**	10875322		
	**1,25**	**0,01687**	10939899		
	**1,25**	**0,00338**	10921268		
	**1,25**	**0,02889**	10833416		
**Fmo1**	**1,25**	**0,00437**	10769361	M84719	flavin containing monooxygenase 1
**Dhx15**	**1,25**	**0,00299**	10772967		DEAH (Asp-Glu-Ala-His) box polypeptide 15
	**1,25**	**0,00357**	10756663		
	**1,25**	**0,00741**	10784101		
	**1,25**	**0,01106**	10939958		
	**1,25**	**0,01793**	10708538		
**Zinki**	**1,25**	**0,00382**	10759488	DQ490055	Arg3.1/Arc mRNA-binding zinc finger protein
	**1,25**	**0,00438**	10863469		
**RGD1306 583**	**1,25**	**0,01256**	10821134	BC088172	similar to RIKEN cDNA 2410002O22 gene
	**1,25**	**0,00335**	10746652		
	**1,25**	**0,00312**	10920743		
**Lifr**	**1,25**	**0,01196**	10821741	D86345	leukemia inhibitory factor receptor alpha
**Arap2**	**1,25**	**0,02860**	10772863		ArfGAP with RhoGAP domain, ankyrin repeat and PH domain 2
**RGD1560 584**	**1,25**	**0,02105**	10855948		similar to High mobility group protein 1 (HMG-1)
**Nck1**	**1,25**	**0,01647**	10919548	BC167009	NCK adaptor protein 1
	**1,25**	**0,00300**	10719074		
**Spcs3**	**1,25**	**0,03631**	10788053		signal peptidase complex subunit 3 homolog (S. cerevisiae)
**RGD1560 191**	**1,25**	**0,00454**	10733067		similar to Zinc finger protein 62 homolog (Zfp-62) (ZT3)
**Zinki**	**1,25**	**0,00344**	10759846	DQ490055	Arg3.1/Arc mRNA-binding zinc finger protein
**Nudcd1**	**1,25**	**0,00293**	10903562	BC169014	NudC domain containing 1
**Mettl9|M ettl9b**	**1,25**	**0,02188**	10916804		methyltransferase like 9 | methyltransferase like 9b
**Smek1**	**1,25**	**0,01022**	10891719		SMEK homolog 1, suppressor of mek1 (Dictyostelium)
**Rpa3**	**1,25**	**0,01189**	10762981		replication protein A3
**Inpp5f**	**1,25**	**0,02948**	10711410		inositol polyphosphate-5-phosphatase F
	**1,25**	**0,01035**	10773057		
**Erlec1**	**1,25**	**0,00290**	10778897		endoplasmic reticulum lectin 1
**Cited2**	**1,25**	**0,00264**	10701846	BC087005	Cbp/p300-interacting transactivator, with Glu/Asp-rich carboxy-terminal domain, 2
	**1,25**	**0,03162**	10791602		
**Hat1**	**1,25**	**0,02530**	10836815	BC087663	histone acetyltransferase 1
	**1,25**	**0,00918**	10832081		
	**1,25**	**0,00094**	10869047		
	**1,25**	**0,00438**	10890017		
**Fam134b**	**1,25**	**0,02149**	10813949		family with sequence similarity 134, member B
**Crls1**	**1,25**	**0,02571**	10840131	BC085849	cardiolipin synthase 1
**LOC6880 19**	**1,25**	**0,01794**	10902409		similar to THAP domain containing, apoptosis associated protein 2

Table S3. Primers for gene expression analysis by quantitative real-time PCR (qRT-PCR).

qPCR PRIMERS

Gene	Forward primer (5'-3')	Reverse primer (5'-3')	Product size (bp)
Hprt	TTGTTGTTGGATATGCCCTTGA	GGCTTTTCCACTTTCGCTGAT	83
Gusb	CATCGGAGAGCTCATCTGGAA	CTGCCATCTTGGGGTTTCTCT	113
Tbp (Taf9l)	AAGATTTGCCGTGCAGATTCC	TGAGGAAACCATGTTGGTGGT	142
Calb2	CGCCCTCCTGAAGGATCTGTA	GGCTCACTGCAAAGCACAATC	144
Chrm2	TCTCCAAGTCTGGTGCAAGGA	GAGCCTTGCCATTCTGGATCT	103
Chrna3	GCCGAAAACATGAAAGCACAG	CCGTTCCTAAAATGCACACCA	121
Crabp1	GGATGGCCCCAAAACTTACTG	CTTGTGCACACCACATCATCG	87
Dpp4	CCCAACTCCAGAGGACAACCT	GGGCTTTGGAGATCTGAGCTG	149
Esr1	CTTCTGGAGTGTGCCTGGTTG	CAAAGATCTCCACCATGCCTTC	145
Gal	GAACAGCGCTGGCTACCTTCT	AACTCCCTCTTGCCTGTGAGG	90
Galr1	CTGCCCTTACTGCTCATCTGCT	GCCAAATACCACAACGACCAC	141
Htr3a	CCTTCCAAGCCAACAAGACTG	TTGGTGGTGGAAGAGGGCTAT	120
Isl1	TCAGCCTGCTTTTCAGCAACT	GGACTGGCTACCATGCTGTTG	126
Mst4	AGAACAATGCAAGCCGAAACC	AGGGGATTCATCCGCAGAAC	143
Nampt	AGGGACCTTTGTCACCCTTGA	TCCATGTTCAGCTGTGCATTTT	144
Nmu	CTGCAGCTCGTTCCTCAACTG	TTGTTGACCTCTTCCCATTGC	127
Nos1	TTAGCAATGACCGAAGCTGGA	GAGCAGCCGAGACTCGTTTTT	114
Npy	GATCCAGCCCTGAGACACTGA	CACCACATGGAAGGGTCTTCA	94
Scg2	ATCGAAGGCTTACCGATTTGG	CTGGTTTCGCTGGAAGGAAG	94
Snap25	CAGAATCGCCAGATTGACAGG	CAGCATCTTTGTTGCACGTTG	90
Sst	ACCCCAGACTCCGTCAGTTTC	CAGGGCATCGTTCTCTGTCTG	122
Uchl1	AATGTGGACGGCCACCTCTAC	TGCAGCAGAGAGTCCTCTGAAC	86

Table S4. Summarized parameters of Sequenom primer pairs designed with Sequenom EpiDesigner.

SEQUENOM PRIMERS

Gene	Forward primer (5'-3')	Reverse primer (5'-3')	Target length (bp)	Target CpG	CpG Analyzed
Gal	GTTTTGGGGTGGTTTATTTTAGTTT	ACTAATTAATACAAATCCAAATATCTCC	472	34	32
Snap25	AGGAAAGGTATAGGTTATTAGGAAAA	AAAACTCTTTACCTTATCTTCTTCAAA	484	20	19
Uchl1	GAAGGTTTAGTTTTTTGTTTTTAAGG	ACTTTTTCTCAATATTTCAAAAACCC	516	20	19
Crabp1	TTATTTAGTTTAAAATAGGATGTGGG	CACCAACTTACCCAAAACCTTAAA	497	40	34
Npy	TGTTTTTTAAGTATAGTGTTTGGTTTTT	CCCAATTAATCCTAACACTCACCA	515	33	32
Dpp4	TATTTGTTGGAGTGGTTTTATGGTT	CAATTAAATCCCAATATACAATATCC	510	35	32
Mst4	GTTGGGATTTGGGTTTTAGTGAT	ACCAAACAAACTTTTACAACTCCAA	485	27	24
Isl1	GGAGGTAGAAAAGGGAGTTTTTAGTT	CAAATAATAACCCCTAAAATTACCAAA	581	67	66
Gata5	GGGTAGGATTATTGGGTTTTTTTTA	ACAACAAAATTCAAAACTATTCAAA	568	40	37

Supplementary Methods

Animal treatment. *p,p*'-DDE was dissolved in ethanol, with the amount of *p,p*'-DDE administered to each animal corrected (St/DDE and HF/DDE) for water intake and animal average weight. In the control groups, St and HF, the same final volume of ethanol (0.01%) was added to the fluid source. The water and chow were supplied ad libitum and every 3-4 days, water and animal pellet food were renewed and the intake assessed. At the end of the 12 weeks of treatment, 4-6 hours fasted animals were anesthetized with a mixture of ketamine (50 mg/kg) and medetomidine (1 mg/kg) and maintained with isoflurane. The body composition of each rat was determined by bioelectrical impedance (Quantum /S bioelectrical impedance analyzer (RJL Systems, Akern SRL, Florence, Italy)), according to the described in the literature [1]. Before perfusion of the vascular compartment with a saline solution (NaCl 0.9%, w/v), blood was drawn from the left ventricle into tubes with or without heparin to obtain plasma and serum, respectively, and fractions were frozen at -80°C until analysis. Organs were dissected, pat dried and weighed before snap freezing in liquid nitrogen and storing at -80°C until further analysis.

Adipocyte isolation and lipolysis assay. Tissues with approximately 5 g were dissected and washed. AT was minced in a collagenase buffer at a 1:1 volume ratio (PBS 1X containing 2% fatty acid-free low endotoxin BSA (bovine serum albumin; Sigma-Aldrich, St Louis, MO) and 250 U/mg collagenase A (Roche Diagnostics, Basel, Switzerland)) and incubated at 37ºC for 45 min in a water-bath shaker at 80 oscillations/min. Once digestion was complete, the product was filtered with a normal sterile cell strainer, followed by an additional filtration with a 150-µm nylon mesh (Sefar, Heiden, Switzerland) and a centrifugation at 100 x g for 1 min. The layer of floating cells was then washed 3x with 10% (v/v) Krebs-Ringer bicarbonate buffer with HEPES (KRBH) at 37°C and finally suspended in KRBH for 1 h before the lipolysis assay. To begin basal lipolysis measurements, a total of 800 µL of adipocyte suspension was added to vials with 4 mL of KRBH buffer containing 0.5% fatty acid-free BSA. While stirring, 100 µL of cell suspension was added to each test tube and incubated at 37ºC for 90 min with mild agitation. After incubation, the tubes were placed on ice to halt the incubation. Lipolysis was evaluated by measuring the free glycerol using Free Glycerol Reagent (Sigma F6428) and NEFAs quantified using an NEFA-HR(2) assay (Wako

Chemicals GmbH, Neuss, Germany). The lipid content was quantified by Dole extraction to normalize the lipolysis results [2].

1 Smith, D., Jr., Johnson, M. & Nagy, T. Precision and accuracy of bioimpedance spectroscopy for determination of in vivo body composition in rats. *Int J Body Compos Res* **7**, 21-26 (2009).

2 Ge, H. *et al.* Activation of G protein-coupled receptor 43 in adipocytes leads to inhibition of lipolysis and suppression of plasma free fatty acids. *Endocrinology* **149**, 4519-4526, doi:10.1210/en.2008-0059 (2008).
